# Metabolomic profiling in tomato reveals diel compositional changes in fruit affected by source–sink relationships

**DOI:** 10.1093/jxb/erv151

**Published:** 2015-04-11

**Authors:** Camille Bénard, Stéphane Bernillon, Benoît Biais, Sonia Osorio, Mickaël Maucourt, Patricia Ballias, Catherine Deborde, Sophie Colombié, Cécile Cabasson, Daniel Jacob, Gilles Vercambre, Hélène Gautier, Dominique Rolin, Michel Génard, Alisdair R. Fernie, Yves Gibon, Annick Moing

**Affiliations:** ^1^INRA, UR1115 Plantes et Systèmes de culture Horticoles, Domaine St Paul, Site Agroparc, 84914 Avignon, France; ^2^INRA, UMR1332, Biologie du Fruit et Pathologie, 71 av Edouard Bourlaux, 33140 Villenave d’Ornon, France; ^3^Plateforme Métabolome du Centre de Génomique Fonctionnelle Bordeaux, MetaboHUB, IBVM, Centre INRA Bordeaux, 71 av Edouard Bourlaux, 33140 Villenave d’Ornon, France; ^4^Max-Planck-Institut für Molekulare Pflanzenphysiologie, Am Mühlenberg 1, 14476 Potsdam-Golm, Germany; ^5^Instituto de Hortofruticultura Subtropical y Mediterranea (IHSM), Universidad de Málaga-Consejo Superior de Investigaciones Científicas, Departamento de Biología Molecular y Bioquímica, Málaga, Spain; ^6^Univ. Bordeaux, UMR1332, Biologie du Fruit et Pathologie, 71 av Edouard Bourlaux, 33140 Villenave d’Ornon, France

**Keywords:** Diurnal changes, fruit metabolism, ^1^H-NMR, MS, metabolomics, *Solanum lycopersicum*.

## Abstract

A detailed study of the diurnal compositional changes was performed in tomato (*Solanum lycopersicum* cv. Moneymaker) leaves and fruits. Plants were cultivated in a commercial greenhouse under two growth conditions: control and shaded. Expanding fruits and the closest mature leaves were harvested during two different day/night cycles (cloudy or sunny day). High-throughput robotized biochemical phenotyping of major compounds, as well as proton nuclear magnetic resonance and mass spectrometry metabolomic profiling, were used to measure the contents of about 70 metabolites in the leaves and 60 metabolites in the fruits, in parallel with ecophysiological measurements. Metabolite data were processed using multivariate, univariate, or clustering analyses and correlation networks. The shaded carbon-limited plants adjusted their leaf area, decreased their sink carbon demand and showed subtle compositional modifications. For source leaves, several metabolites varied along a diel cycle, including those directly linked to photosynthesis and photorespiration. These metabolites peaked at midday in both conditions and diel cycles as expected. However, transitory carbon storage was limited in tomato leaves. In fruits, fewer metabolites showed diel fluctuations, which were also of lower amplitude. Several organic acids were among the fluctuating metabolites. Diel patterns observed in leaves and especially in fruits differed between the cloudy and sunny days, and between the two conditions. Relationships between compositional changes in leaves and fruits are in agreement with the fact that several metabolic processes of the fruit appeared linked to its momentary supply of sucrose.

## Highlight

Using metabolomics in tomato, we confirmed the existence of diel patterns in leaf composition and showed lower but significant diel changes in expanding fruit depending on the potential carbon supply.

## Introduction

Source-to-sink relationships are central for growth and performance in plants (Lemoine *et al.*, 2013; Ruan *et al.*, 2013), especially fruit crops. After fruit set, fruit growth and development depend largely on the import of metabolites, mineral elements, and water from other organs. Most metabolites are imported from photosynthetic leaves, but fruit photosynthesis also contributes to carbon nutrition, especially in young fruits (Lytovchenko *et al.*, 2011). The study of source–sink relationships, and especially assimilate transport and partitioning into competing organs, is therefore of special interest in relation to the improvement of fruit yield and quality (Ho, 1996). For fleshy fruits, source–sink relationships have been studied using a range of approaches, such as whole-plant physiology including measurements of biomass allocation and modelling (Heuvelink, 1995), labelling experiments (Minchin *et al.*, 1997), photosynthetic rates and carbohydrate levels (Blanke, 2009), measurements of enzyme activities in fruit (Wang *et al.*, 1993), genetics (Yelle *et al.*, 1991), ecophysiological modelling (Liu *et al.*, 2007), and, more recently, transcriptomics (Pastore *et al.*, 2011). Moreover, systems biology is an emerging approach for source and sink studies.

In source–sink studies, the source and sink balance is often modified by changing alternatively the source or the sink. The photosynthesizing source providing carbon can be modified using for instance changes in light intensity or duration (Vasseur *et al.*, 2011) or leaf thinning (Arnold *et al.*, 2004), and/or the fruit sink utilizing carbon can be modified through fruit thinning (Do *et al.*, 2010). Irrespective of the experimental design and approach chosen for their study, source–sink relationships depend on the diurnal behaviour of source leaves, i.e. exporting leaves.

Diurnal changes in the biochemical composition of mature leaves have been investigated intensively, for example in *Arabidopsis* (Gibon *et al.*, 2006) and potato (Urbanczyk-Wochniak *et al.*, 2005). Synchronization of leaf metabolism with diel environmental changes contributes to the regulation of plant growth and increases in plant fitness (Harmer, 2009). During the day, photosynthesis in mature leaves fuels carbohydrate synthesis and sucrose export to the growing vegetative or reproductive sink organs. At night, remobilization of starch stored during the day contributes to maintain sucrose export. Diel changes also occur for nitrogen metabolism including nitrate assimilation in leaves (Scheible *et al.*, 2000). While the growth rate of fleshy fruit has been shown to vary diurnally in tomato (Guichard *et al.*, 2005), in relation to water potential variations, very few studies describe diel compositional changes in fruits. A work on apple fruit during the growing phase revealed no changes in sugar content (Klages *et al.*, 2001). An earlier work on tomato fruit during the expansion phase showed no significant changes in hexose and malate content (Pearce *et al.*, 1992).

Nowadays, leaf and fruit compositional changes can be described in detail using metabolomics combining several analytical strategies (Hall, 2011). Gas chromatography coupled with mass spectrometry (GC-MS) and proton nuclear magnetic resonance spectroscopy (^1^H-NMR) of polar extracts give access to a range of primary metabolites. Liquid chromatography coupled with mass spectrometry (LC-MS) of semi-polar extracts provides relative quantification of secondary metabolites belonging to several families of compounds including flavonoids, hydroxycinnamates, and glycoalkaloids. Such analytical approaches have largely been used recently for crop species including tomato (de Vos *et al.*, 2011). However, source–sink studies involving metabolomics remain rare for fruit crops. As most works about changes in metabolites in source–sink interactions have been derived from *Arabidopsis*, it is now meaningful to study crop species of economic importance such as tomato plants that have multiple fruits that serve as strong sinks.

In the present work, diurnal compositional changes were measured in greenhouse-grown tomato expanding fruits and the closest mature leaves using a combination of metabolomics approaches based on NMR and MS, and on robotized microplate measurements of starch, proteins, and total free amino acids. Since shading has been shown to affect both yield and fruit quality in tomato (Gent, 2007), we also investigated the leaves and fruits of plants experiencing different light regimes (cloudy vs sunny day, and control vs shading condition). With contrasted carbon availability at the plant level, leaf and fruit diel variations were investigated to study the relationships between the composition of the mature leaves close to the harvested fruit truss and that of the fruit pericarp using metabolite networks. These approaches provided information about possible metabolic regulations in the context of the relationships between source leaf and sink fruit.

## Materials and methods

### Plant material and growth conditions

Tomato (*Solanum lycopersicum* L. cv. Moneymaker) plants were grown in a greenhouse in south-west France from June to September according to commercial practices as detailed in Supplementary text at *JXB* online. Fruit load was set at six fruits per truss when needed. The entire fruit development from anthesis to the red-ripe stage lasted about 55 d. Two conditions were applied: ‘control’ (276 plants), and low-light conditions, referred to as ‘shaded’ [138 plants with a shadow net stopping 60% of incident light, with limited effects on temperature, installed in early July when fruits of truss 3 were at about 6 d post-anthesis (DPA)].

During the plant culture, we focused on two diel cycles hereafter referred to as ‘Experiments’. Experiment 1 (Exp. 1) was performed in late July on an overcast day, with expanding fruits located on truss 3 (23±1 DPA), and Exp. 2 was performed in late August with a clear sky, with expanding fruits located on truss 8 (24±1 DPA). For Exp. 1, the shaded condition lasted about 2 weeks. For Exp. 2, in which the shaded condition lasted about 9 weeks, several measurements, including those issued from a destructive harvest, were studied in more detail.

### Ecophysiological measurements

Photosynthetically active radiation (PAR) and temperatures were measured using a PAR Quantum Sensor (LI-190; LICOR, Lincoln, NE, USA) and a resistance temperature detector (PT-100) located at the top of the canopy. During Exp. 2, the temperature of four growing fruits per condition was measured using thermocouples (type K).

Plant development was followed weekly by measuring the plant height and leaf number on nine plants per condition. In addition, destructive descriptions of six plants per condition were carried out 1 week before Exp. 2. This provided measurements of the leaf area, fruit load, and plant aerial biomass dry weight (DW, oven drying at 70 °C). For the leaves closest to truss 8, the leaf area was estimated using digital photography and image analysis (ImageJ software, http://imagej.nih.gov/ij/), the specific leaf area (SLA) was determined, and the potential net photosynthetic rate was calculated using the leaf area and an estimation of radiation interception as described previously (Baldazzi *et al.*, 2013).

Fruit growth was followed through measurement of fresh weight (FW) and dry matter content (oven drying) of entire fruits harvested on trusses 5, 6, and 7, with about 15 fruits per stage along fruit development. Growth rate was calculated using mean fruit DW curves modelled using a logistic function. Rates of fruit respiration and carbon consumption were calculated using an ecophysiological process-based model (Jones *et al.*, 1991; Colombié *et al.*, 2015) as detailed in Supplementary text. For Exp. 2, absolute levels of major metabolites in the pericarp were summed and converted into moles of carbon, which allowed calculation of the corresponding diel amplitude of non-structural carbon content on a DW or organ basis.

### Sampling

Expanding fruits (23 or 24±1 DPA) and leaves were harvested every 4h during the diel cycle, with four biological replicates. A fruit biological replicate was made up of four to five individual fruits from different plants. Fruits were weighed and a quarter of pericarp of the equatorial zone was selected. A leaf biological replicate was made up of portions of the mature leaves close to the harvested fruit truss. The fruit or leaf samples were frozen immediately in liquid nitrogen. Samples were then ground in liquid nitrogen and stored at –80 °C until analysis. Freshly frozen powder was used for robotized assays and GC-MS analyses, and lyophilized powder for ^1^H-NMR and LC-MS analyses, and to determine dry matter contents.

### Robotized analyses of metabolites and proteins

Twenty milligrams of FW was extracted with ethanol/water, and analysed using microplates as described previously (Biais *et al.*, 2014). The sum of free amino acids was determined in the supernatant, and the starch (in glucose equivalent) and protein contents were determined in the pellet. Details are given in Supplementary text.

### ^1^H-NMR analyses

For ^1^H-NMR profiling, polar metabolites were extracted and analysed from 20mg of DW as described previously (Biais *et al.*, 2009) with minor modifications as detailed in Supplementary text. For absolute quantification of metabolites, calibration curves were prepared and analysed under the same conditions. Metabolite concentrations were calculated using AMIX software (version 3.9.7; Bruker, Karlsruhe, Germany) and the calibration curve data, and converted to contents expressed on a DW basis. The ^1^H-NMR spectra were converted into JCAMP-DX format and have been deposited, with associated metadata, in the Metabolomics Repository of Bordeaux MeRy-B (Ferry-Dumazet *et al.*, 2011; http://www.cbib.u-bordeaux2.fr/MERYB/projects/home.php?R=0&project_id=47).

### LC-quadrupole time-of-flight (Q-TOF)-MS analyses

For LC-MS analyses, 20mg of DW was extracted and analysed as described previously (Pascual *et al.*, 2013) with minor modifications as detailed in Supplementary text. Nineteen compounds were targeted, 16 of which were putatively identified according to their exact masses and the literature (Mintz-Oron *et al.*, 2008; Gómez-Romero *et al.*, 2010). Sample compound areas were normalized with the internal standard area and with the compound area mean of quality-control samples, providing relative quantification data.

### GC-TOF-MS analyses

Metabolites for GC-TOF-MS were extracted from 30mg of FW and analysed as described previously (Osorio *et al.*, 2012) with minor modifications as detailed in Supplementary text. Chromatograms and mass spectra were evaluated using Chroma TOF 1.6 and TagFinder 4.0 software (Luedemann *et al.*, 2008). Quantities of metabolites are expressed as relative intensity, based on peak integration, and relative to the internal standard.

### Statistical analyses

Metabolite contents expressed on a DW basis were used for statistical analyses. Principal component analysis (PCA, correlation matrix) was performed using R scripts in the BioStatFlow web application (http://bit.ly//biostatflow). One-factor analysis of variance (ANOVA) was performed using MultiExperiment Viewer, version 4.8 (Saeed *et al.*, 2003). K-means clustering (MultiExperiment Viewer version 4.8) was performed on the means of the biological replicates (data mean centred and reduced to unit variance, Pearson correlation distance) after ANOVA filtering (*P*<0.05). To visualize co-regulations between leaf and fruit compositional changes, partial correlation graphs were used. We chose a Gaussian graphical model (GGM) approach based on pairwise Pearson correlation coefficients conditioned against the correlation of all other metabolites, since GGMs are much sparser than total correlation networks (Krumsiek *et al.*, 2011). Network cartography was done on log_2_-transformed data, with false discovery rate correction (*q*<0.001), and Fruchterman layout, using R scripts in BioStatFlow (NETGRAPH; Opgen-Rhein and Strimmer, 2007) and Cytoscape software version 3.0 (Shannon *et al.*, 2002; http://www.cytoscape.org/).

## Results

During the tomato plant culture, we studied two experimental days that had similar sums of temperatures but different sums of irradiance. The 24h cumulative PAR over the diel cycle was 9.18 and 26.13mol photons m^–2^ for the control condition, and 4.15 and 8.21mol photons m^–2^ for the shaded condition, for Exps 1 and 2, respectively.

### Plant phenotypes are affected after long-term shading

The effect of shading on plant development was marked (Table 1). Several measurements performed only for Exp. 2, with shaded plants grown with a longer shading period, revealed that these plants were etiolated, as plant height and SLA were significantly higher and the leaf number significantly lower (Table 1). The biomass of the vegetative aerial parts (leaves and stems) was significantly lower for the shaded condition (Table 1). Moreover, the plants allocated about 5% more of their aerial vegetative biomass to the leaves under shading (70% compared with 65% in the control condition).

**Table 1.** Effect of shading on tomato plant development at the time of each experiment Mean±SD (*n*=9 for plant height and leaf number, *n*=6 for plant biomasses, *n*=12 for leaf area and SLA, *n*>80 for fruit weight). The individual leaf area and SLA were measured for the two leaves closest to truss 8. For each experiment, * indicates a significant difference between the shaded and control conditions (Student’s *t*-test, *P*<0.05).

	Exp. 1		Exp. 2	
	Control	Shaded	Control	Shaded
Plant height (cm)	146.7±6.7	143.6±14.3	231.7±15.3	265.8±12.9*
Leaf number per plant	28.6±1.7	24.7±3.7*	44.3±2.7	40.1±1.3*
Total aerial vegetative biomass (g DW per plant)	–	–	173.4±10.8	101.9±9.1*
Total leaf biomass (g DW per plant)	–	–	113.0±7.1	70.5±6.3*
Total stem biomass (g DW per plant)	–	–	60.4±6.8	31.4±3.4*
Total fruit biomass (g DW per plant)	–	–	140.2±21.4	56.8±12.7*
Individual leaf area (cm^2^ per leaf)	–	–	204.0±63.4	214.8±68.7
SLA (cm^2^ g^–1^ DW)	–	–	129.6±9.8	287.7±51.2*
Fruit number on truss 8	–	–	5.17±1.34	2.00±1.63*
Harvested fruit weight				
g FW per fruit	41.0±10.3	33.0±8.3*	46.2±9.1	20.7±7.9*
g DW per fruit	2.52±0.64	1.85±0.44*	2.83±0.56	1.16±0.44*

Total fruit biomass was significantly lower under shading. In addition, the fruit number of truss 8 was low (Table 1), due to low flower initiation and/or high flower or fruit abortion observed. For Exp. 2, mean fruit temperatures were slightly higher under control conditions but only from 07:00 to 17:30h (Supplementary Fig. S1 at *JXB* online). The mean fruit temperature over the diel cycle was 20.8±0.3 and 20.2±0.2 °C (*n*=4) for the control and shaded conditions, respectively. Therefore, we worked on two diel cycles with contrasted skies (overcast vs clear), and with two different irradiance levels (control vs shaded, with limited fruit temperature differences). Although fruits under expansion were harvested at the same age, the average fruit weight was significantly lower in the shaded (33.0 and 20.7g of FW, or 1.9 and 1.2g of DW) than control condition (41.0 and 46.2g of FW, or 2.5 and 2.8g of DW) for Exp. 1 and Exp. 2, respectively (Table 1). This corresponded approximately to 44 and 50% of the fruit FW at red-ripe stage for control and shaded conditions, respectively.

### Fruit composition is less affected by the experiment, condition, and harvest time compared with the leaf

**Fig. 1.**
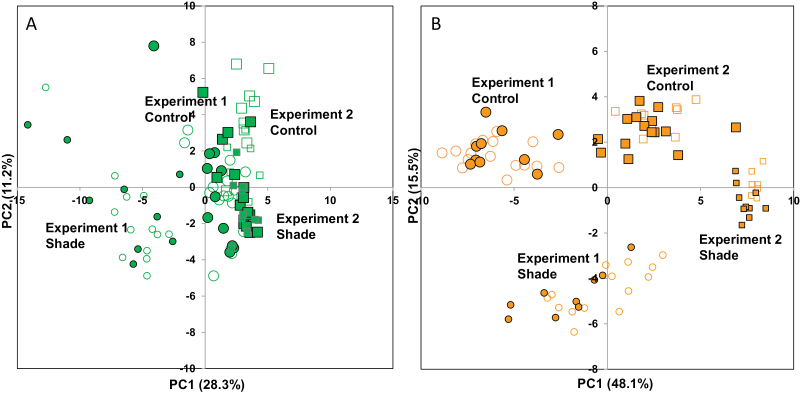
PCA in tomato mature leaf or expanding fruit (23 or 24±1 DPA) in Exp. 1 (2 week of shading) and Exp. 2 (9 weeks of shading) under the control or shaded condition across a day/night cycle. Scores plots of the first two PCs. Exp. 1, circles; Exp. 2, squares. Larger symbols, control condition; smaller symbols, shaded condition. Open symbols, day period; closed symbols, night period. (A) PCA of 70 compounds in tomato leaf. (B) PCA of 56 compounds in tomato fruit. (This figure is available in colour at *JXB* online.)

Complementary analytical techniques for biochemical profiling provided data on 70 compounds in leaves and 56 compounds in fruits (Supplementary Tables S1–3 and Fig. S3 at *JXB* online). To visualize leaf or fruit data, we performed PCA for the two experiments and conditions. Leaf samples harvested during the day and night periods were not clearly separated on the PC1×PC2 plan (Fig. 1A). The PCA scores plot revealed that leaf composition was similar in the two experiments for the control condition and hardly distinguishable from that of the shaded condition in Exp. 2. However, the leaves of the shaded condition in Exp. 1 clearly separated from all other samples along PC1. Since the shading condition was applied in early July, the closeness of the control and shaded leaf samples in Exp. 2 may express better acclimation of the shaded plants in Exp. 2 performed in August than in Exp. 1 performed in July.

The first two dimensions resumed a higher part of total inertia for the fruit PCA (Fig. 1B, 64%) than the leaf PCA (Fig. 1A, 40%). For fruit, examination of the PCA scores plot (Fig. 1B) revealed a clear separation of the experiments as well as conditions but a parallel effect of shading in Exp. 1 and Exp. 2. Fruits harvested during the day and night periods were not clearly separated. Overall, according to the percentage of total variability explained by PC1 and PC2, the global compositional changes induced by shading in fruit in one experiment seemed lower than the changes between experiments for a given condition. The global changes between experiments may be related to changes in the plant phenology and local or greenhouse climate.

ANOVAs showed that 73% of the compounds were affected by the harvest time in at least one condition of one experiment in leaf, and 63% in fruit. In order to visualize the diurnal variations, the coefficient of variation (CV) of each compound was calculated based on its content means at each harvest time for each condition (Supplementary Fig. S2 at *JXB* online). The highest CVs reached about 80% in leaf and only 35% in fruit. The amplitudes of these variations in fruit were compared with those in leaf using the CV medians (Table 2). The medians in fruit were about half those in leaf.

**Table 2.** Variability of compound contents during a diel cycle in tomato CV median±SD of 70 metabolites measured in leaf, or 56 metabolites in fruit, harvested at seven times, except for the shaded condition of Exp. 2 with five time points (*n*=4).

	Exp. 1		Exp. 2	
	Control	Shaded	Control	Shaded
Mature leaf	21.4±10.8	15.9±15.4	18.3±15.4	18.5±14.5
Expanding fruit	9.6±3.5	8.9±6.2	6.3±6.2	8.3±4.3

### Leaf diel patterns are conserved for a subset of metabolites or depend on the environment for most other metabolites

**Fig. 2.**
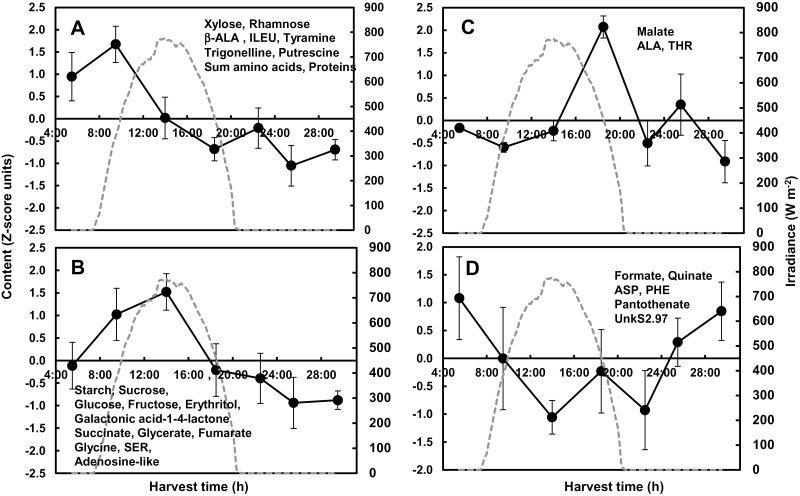
Diel patterns of 30 compounds in tomato mature leaf, across a day/night cycle for the control condition of Exp. 2. Four patterns were inferred through K-means clustering after ANOVA filtering. Solid-line graphs indicate the mean of all compounds in each cluster. Vertical bars represent the standard deviation. The name of compounds in each cluster is reported. The dashed line indicates the irradiance outside the greenhouse.

**Fig. 3.**
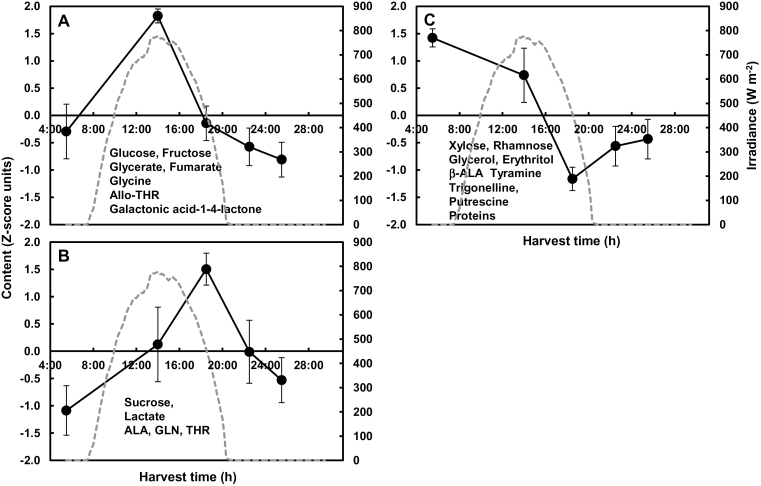
Diel patterns of 21 compounds in tomato mature leaf, across a day/night cycle for the shaded condition of Exp. 2. Three patterns were inferred through K-means clustering after ANOVA filtering. See Fig. 2 legend for further details.

Clear diel patterns appeared in mature leaves for a range of compounds spanning different compound families (shown as heat maps, Supplementary Fig. S3A–D). These patterns were classified using K-means clustering after ANOVA filtering. For the control condition of Exp. 2, 32 of the 70 compounds were clustered into four groups. The first cluster (Fig. 2A) peaked in the morning and comprised two minor sugars, five amino compounds, the sum of free amino acids, and protein content. The second one (Fig. 2B) peaked at midday; it contained several intermediates of photorespiration, and several major carbohydrates in relation to photosynthesis, and four other compounds. The third one (Fig. 2C) peaked early in the afternoon and included malate and two amino acids. The fourth cluster (Fig. 2D) contained two organic acids and two amino acids, rising at night. For the shaded condition of Exp. 2, starch did not accumulate during the light period, but 21 other compounds showed a significant time effect. Among the latter compounds, 18 were common with the control condition. The 21 compounds were clustered into three groups. The first cluster (Fig. 3A) peaked at midday and included two intermediates of photorespiration, hexoses, and one organic acid or amino acid. The second one (Fig. 3B) peaked in the afternoon; it contained sucrose, one organic acid, and three amino acids. The third one (Fig. 3C) peaked at the end of night and included four alcohols or minor sugars, four amino compounds, and protein content.

In Exp. 2, none of the leaf hydroxycinnamate, flavonoid, or glycoalkaloid content was affected by harvest time. In Exp. 1, 30 and 32 compounds showed a significant time effect in the control and shaded conditions, respectively, with 22 common compounds (Supplementary Fig. S3A, B). Diurnal leaf patterns appeared for secondary metabolites during Exp. 1. Among these metabolites, α-tomatine, dehydrotomatine, and chlorogenate had a lower content at midday in both conditions (Supplementary Fig. S3A, B). It was noteworthy that several compounds including those directly related to photorespiration and photosynthesis maintained similar diurnal patterns in all conditions. They included sucrose, rhamnose, erythritol, glycerate, alanine, β-alanine, glycine, tyramine, and trigonelline, and also proteins (Supplementary Fig. S3A–D).

### Expanding fruit presents diel metabolite patterns that depend on the environment

**Fig. 4.**
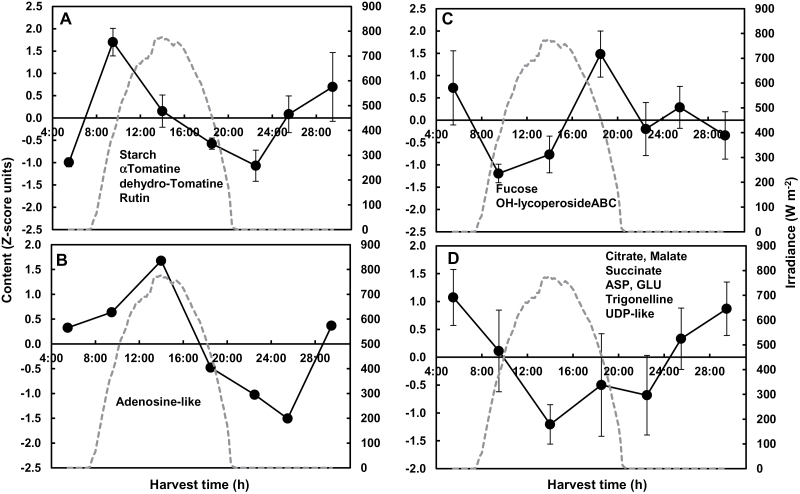
Diel patterns of 14 compounds in expanding tomato fruit (24 DPA) across a day/night cycle for the control condition of Exp. 2. Four patterns were inferred through K-means clustering after ANOVA filtering. See Fig. 2 legend for further details.

**Fig. 5.**
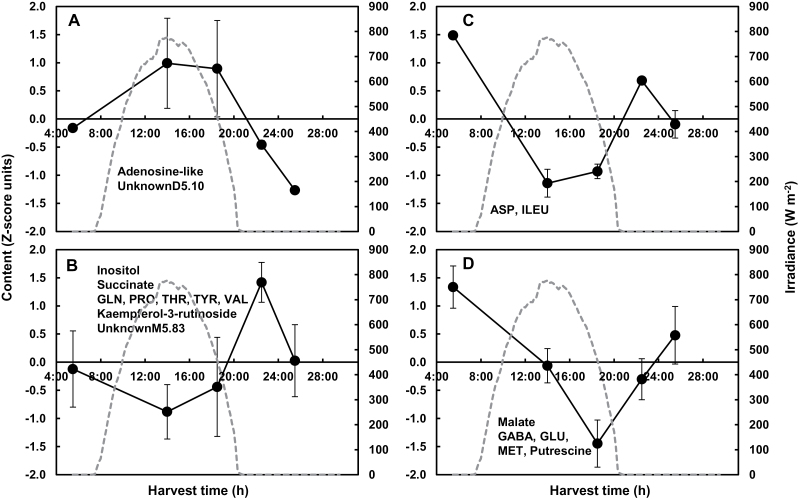
Diel patterns of 18 compounds in expanding tomato fruit (24 DPA) across a day/night cycle for the shaded condition of Exp. 2. Four patterns were inferred through K-means clustering after ANOVA filtering. See Fig. 2 legend for further details.

Diel patterns appeared in fruits for several compounds of different families (heat maps, Supplementary Fig. S3E–H) and were classified into groups for each condition. For Exp. 2, 14 and 18 out of 56 compounds showed a significant time effect in the control and shaded conditions, respectively, with five common compounds. For the control condition of Exp. 2, the 14 compounds were clustered into four groups (Fig. 4). The first cluster (Fig. 4A) peaking early in the morning comprised starch and three secondary metabolites. The second cluster (Fig. 4B) comprised only an adenosine-like compound, peaking at midday. The third cluster (Fig. 4C), peaking at the end of the day, comprised a minor sugar and a glycoalkaloid. The fourth cluster (Fig. 4D) included three TCA cycle intermediates and two major amino acids, with higher contents at night and lower contents in the light. For the shaded condition, the 18 compounds affected by harvest time were clustered into four groups (Fig. 5). The first cluster (Fig. 5A) peaked in the afternoon and included an adenosine-like compound. The second one (Fig. 5B) peaked at the end of day and contained inositol, succinate, five amino acids, and one flavonoid. The third one (Fig. 5C) peaked at the end of the night and included two amino acids. The fourth cluster (Fig. 5D) included malate and four amino compounds, which increased at night and decreased during the day. For Exp. 2, several compounds showed close, although not fully similar, trends in both conditions, including an adenosine-like compound, malate, succinate, aspartate, and glutamate. The diurnal changes in the latter four compounds, determined in absolute content values, are presented in Supplementary Fig. S4 at *JXB* online. For aspartate and glutamate, the amplitude of the diel variation was higher in the control than the shaded condition. The diurnal changes of several other compounds of importance for source–sink relationships (sucrose, starch, amino acids, and proteins) are presented in Supplementary Fig. S5 at *JXB* online. No significant time effects were observed, except for starch in the control condition and the sum of amino acids in the shaded condition.

For Exp. 1, a lower number of compounds showed a significant time effect than for Exp. 2. Only four and seven fruit compounds showed a significant time effect in the control and shaded conditions, respectively, with only one common compound between these two conditions, adenosine-like compound (Supplementary Fig. S3E, F). Only malate and adenosine-like compound maintained a similar diurnal pattern in the control condition of both experiments. Only the adenosine-like compound maintained a similar diel pattern in the shaded condition of both experiments.

The patterns of a compound with a significant harvest time effect in both organs could be compared. For the control condition of Exp. 2, adenosine-like compound and aspartate had similar patterns in leaf and fruit, and starch showed a close pattern with a shift, whereas succinate and malate tended to have opposite patterns in the two organs (Figs 2 and 4). For the shaded condition, putrescine followed the same pattern, and glutamine and threonine had similar patterns with delayed peaking in fruit compared with leaf (Figs 3 and 5). For Exp. 1, in the control condition, malate also tended to have opposite patterns in fruit compared with leaf (Supplementary Fig. S3A, E). Moreover, a range of amino acids tended to peak later in leaf and earlier in fruit in the shaded compared with the control condition (Supplementary Fig. S3A, B, E, F).

### Diel amplitudes in compositional changes are observed for leaf and fruit

The major metabolites, including soluble sugars, organic and amino acids, and starch, quantified in absolute values presented a total content of 531mg g^–1^ of DW in leaf and 528mg g^–1^ of DW in fruit (mean of all experiments and conditions). For Exp. 2, the diurnal amplitudes of non-structural carbon (C) content in leaves were calculated from the contents of these compounds (Table 3). The daily leaf cumulated potential net photosynthesis was slightly higher for the control (14 mmol of C per leaf d^–1^) than the shaded condition (11 mmol of C per leaf d^–1^), the lower photosynthesis rate in the shaded condition being partly compensated by the larger SLA (Table 1). The daily carbon consumption rate of the fruits of plants in the shaded condition (Table 1) was about half that in the control condition: 5 versus 9 mmol of C per fruit d^–1^, respectively. A putative maximum number of expanding fruits that can be sustained by one leaf (considering leaf respiration at night as negligible) was then calculated, based on the estimates of the daily leaf cumulated potential net photosynthesis divided by the daily fruit carbon consumption rate in Table 3. Interestingly, this maximum fruit number per leaf was slightly higher for the shaded condition (about 2.2) than the control condition (about 1.6). However, fruits were also smaller under shading (Table 1). This also means that to sustain all truss 8 fruits (Table 1, about five fruits for control and two fruits for shaded), the contribution of about three leaves would be needed for the control condition but only one for the shaded condition.

**Table 3.** Diel amplitudes of carbon content in leaf and fruit and estimation of carbon balance during Exp. 2 for the control and shaded conditions Photosynthesis and carbon content amplitudes calculated in the leaf close to the fruit truss and an expanding fruit. Leaf potential photosynthesis estimated using leaf area from Table 1. Fruit growth rate calculated from fruit DW changes. Fruit carbon consumption rate calculated as the sum of estimated respiration and growth. Diel amplitudes of non-structural carbon contents calculated from the major metabolites quantitation and assuming the entire fruit similar to pericarp.

		Control	Shaded
Mature leaf			
	Daily cumulated potential net photosynthesis (mmol C per leaf d^–1^)	14.05	10.60
	Diurnal amplitude of non-structural carbon content (mmol C per leaf)	4.00	0.87
Expanding fruit			
	Diurnal amplitude of non-structural carbon content (mmol C per fruit)	1.60	1.68
	Fruit growth rate (mmol C per fruit d^–1^)	6.07	3.46
	Fruit respiration rate (mmol C per fruit d^–1^)	2.64	1.39
	Fruit carbon consumption rate (mmol C per fruit d^–1^)	8.71	4.84

As expected, the diel amplitude of transitory (non-structural) carbon content in leaves was always lower than the daily cumulated potential net photosynthesis (Table 3). Strikingly, it represented 28% of the daily cumulated potential net photosynthesis under control conditions, and only 8% under shading, the estimated carbon export per leaf being similar between the two conditions (about 10 mmol of C per fruit d^–1^). Diel amplitudes of non-structural carbon contents in fruits (Table 3) ranged between 1.6 for the control condition and 1.7 mmol of C per fruit for the shaded condition, and were lower than those per leaf. They were also lower than the carbon content involved in fruit growth per day.

### Several leaf and fruit diurnal patterns seem to be coordinated

**Fig. 6.**
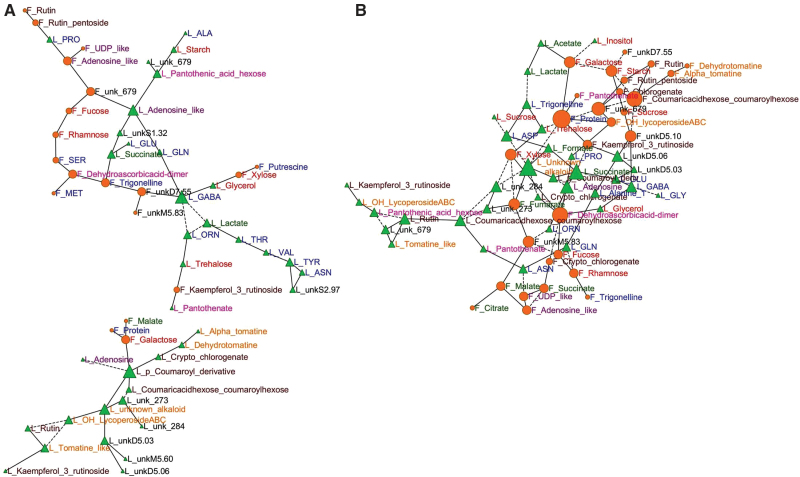
Networks of compounds based on GGM in mature leaf and expanding fruit (23 or 24±1 DPA) for the two experiments. Partial correlations of compounds in fruit or leaf calculated with false discovery rate correction (*q*<0.001) and visualized using Cytoscape. Vertex size is proportional to the number of connections. Vertices are coloured according to the compound family: red, sugars or sugar alcohols; dark green, organic acids; blue, amino compounds; purple, nucleotides/nucleosides; orange, glycoalkaloids; dark brown, phenolic compounds; pink, vitamins or vitamin-related; black, unknown. Triangles, compounds in leaf; Circles, compounds in fruit. Solid vertices, positive correlations; dashed vertices, negative correlations. (A) Control condition; (B) shaded condition.

The links between leaf and fruit metabolic patterns were visualized with networks reconstructed using GGM. Since carbon availability was different in the control and shaded conditions, as highlighted above (Tables 1 and 3), a specific network was reconstructed for each condition. First, the metabolite networks based on the data of the two experiments for the control condition was reconstructed (Fig. 6A). GGM yielded two networks implicating more than two variables, and comprising 56 out of the 126 variables considered, with 60 edges. Each of these two networks comprised compounds from leaf and fruit. The smaller network comprised a majority of leaf secondary metabolites and only three compounds determined in fruit, including malate. The larger network comprised an approximately equivalent number of compounds determined in fruit and leaf. Fifty-three out of the 60 edges corresponded to positive correlations, and only seven edges corresponded to negative correlations. Overall, the compounds appeared to be grouped by organ and compound family with a few exceptions. The compounds with the highest number of connections were leaf γ-aminobutyric acid (GABA) and a leaf *p*-coumaroyl derivative with five connections each. Fourteen compounds could be considered as ‘bridges’ between the two organs, i.e. a compound in one organ linked to another compound in the other organ.

A second metabolite network based on the data of both experiments for the shaded condition was reconstructed. GGM yielded only one large network comprising at least three compounds (Fig. 6B). This network comprised 63 compounds and 92 edges with an approximately equivalent number of compounds determined in fruits and leaves. Sixty-three of the 92 edges corresponded to positive correlations, and 29 edges corresponded to negative correlations. The compounds with the highest number of connections were fruit proteins and a leaf unknown alkaloid, with seven connections each. Compounds tended to group by organ, but the different compound families were more intricate than for the control network. The average number of neighbours was higher in the large cluster obtained with the shaded condition (2.9) than in the two large clusters obtained with the control condition (2.1). Twenty-nine compounds could be considered as ‘bridges’ between the two organs for the large cluster of the shaded condition. Surprisingly, fruit xylose was linked with leaf trehalose. No ‘bridge–compound’ pair was common to the control (Fig. 6A) and shaded (Fig. 6B) condition networks. However, five leaf compounds (trehalose, glycerol, succinate, GABA, and proline) and three fruit compounds (galactose, xylose, and kaempferol-3-rutinoside) were ‘bridge’ compounds in both conditions.

## Discussion

The biochemical composition changed across a diel cycle in mature source leaves and also in expanding green fruits, although in a more limited manner. The metabolites showing a diurnal pattern were influenced by modified irradiance modifying carbon availability. These changes are compared with those in other species and their causes are discussed.

### Diel changes in leaf metabolites depend qualitatively and quantitatively on environment but are preserved for several compounds

Several leaf metabolites with a significant time effect did not exactly recover their initial content after one diel cycle, contrary to *Arabidopsis* grown in a growth chamber (Gibon *et al.*, 2006). This may result from an environmental effect of the preceding day in the greenhouse. However, we had clear diurnal patterns in mature leaf for a range of compounds. As expected, several compounds related to photosynthesis or photorespiration showed a diel pattern in leaves for all conditions and experiments, in agreement with *Arabidopsis* data (Gibon *et al.*, 2006). An early study targeting tomato carbohydrates showed maximum sucrose contents at the end of the light period and overnight starch breakdown (Hammond *et al.*, 1984), in agreement with our results. In a recent GC-MS study of leaves adjacent to a fruiting truss, only serine and threonine underwent significant changes between different harvest moments in the day and night in the Moneymaker variety (Luengwilai *et al.*, 2010). Interestingly, these metabolites also accumulated during the day and decreased at night in the present study. Such a fluctuation is in agreement with the major importance of the photorespiratory pathway for serine biosynthesis in the leaf (Ros *et al.*, 2014) and the fact that serine may act as a metabolic signal for the transcriptional regulation of photorespiration (Timm *et al.*, 2013).

In the present study, leaf tyramine, trigonelline, erythritol, rhamnose, and proteins also showed a diurnal pattern for all conditions and experiments. The role of changes in tyramine and trigonelline is unclear. Rhamnose changes may be related to cell-wall biosynthesis linked to cell enlargement. Indeed, in *Arabidopsis*, a dTDP-d-glucose 4,6-dehydratase homologue predicted to act in rhamnose synthesis peaked at the end of night (Harmer *et al.*, 2000). Proteins peaked in the morning for all conditions and experiments, except the shaded condition of Exp. 1, in agreement with a tight co-ordination of protein synthesis with the momentary supply of carbon as shown for *Arabidopsis* (Pal *et al.*, 2013).

Carbon availability in the leaf, modified through changes in weather or shading, modified the diel patterns. For Exp. 2, sucrose content peaked later in the shaded condition than in the control one, and a starch diurnal pattern was no longer detected in the shaded condition. In addition, diurnal patterns appeared for secondary metabolites in Exp. 1 only, for two glycoalkaloids and a hydroxycinnamate. A range of primary metabolites tended to peak between the end of the night and midday, while the latter secondary metabolites peaked later, towards the end of the day. This different scheduling is in line with the competition for carbon and the view that carbon-based secondary metabolites consist in a sort of overflow for primary carbon metabolism (Aharoni and Galili, 2011). Although α-tomatine and dehydrotomatine belong to nitrogen-based metabolites, they seem to behave like carbon-based secondary metabolites (Royer *et al.*, 2013). The diverse behaviours of secondary metabolites during a diel cycle [e.g. for *Arabidopsis* (Gibon *et al.*, 2006) and the present data] might result from organ, species, or phenology specificities, in relation to their complex functions (Neilson *et al.*, 2013).

### Transient carbon storage remains only marginal in tomato leaves

In the present study, the photosynthetic rates obtained were comparable to those found in *Arabidopsis* (Graf *et al.*, 2010). Leaf starch is usually considered the major form of transient storage of carbon in plants. In *Arabidopsis*, the amount of starch accumulated during the day perfectly matches the needs during the night, and is under the control of both sugar sensing and the circadian clock (Graf *et al.*, 2010). Several studies have shown that *Arabidopsis* leaves respond to decreased light intensity (e.g. Vasseur *et al.*, 2011) by increasing the proportion of carbon fixed by photosynthesis into diurnal starch accumulation, in relation to decreased sink demand. A sudden decrease in sink demand does not influence transient carbon storage in tobacco leaves (Huber and Hanson, 1992) in contrast to what happens in soybean leaves (Rufty and Huber, 1983), which led the latter authors to hypothesize that carbon reserves in other plant parts exert a buffering effect in tobacco. It has been proposed that, under optimal growth conditions, mature tomato leaves export as much carbon as they fix by photosynthesis (Ho, 1978), in line with a report indicating no significant changes in starch and soluble sugars at night (Luengwilai *et al.*, 2010). In our experiments, some carbon remobilization occurred at night in mature leaves under optimal growth conditions but hardly at all under shading. Furthermore, tomato leaves did not increase carbon storage during the day in response to decreased cumulated net photosynthesis, although fruit biomass was more reduced (–59% for an expanding fruit) than cumulated net photosynthesis (–25% for the leaf close to truss 8), reinforcing the idea that most of the carbon storage takes place in other plant parts. Another striking observation is that, under optimal growth conditions, starch but also sucrose and hexoses all peaked towards the middle of the day and began decreasing several hours before dusk. A large proportion of carbon is probably stored in the stem, which constituted about 35% of the dry mass of the plant vegetative aerial part in our study, in the form of sucrose (De Swaef *et al.*, 2013) or starch (Hammond *et al.*, 1984). This calls for detailed investigations of stem metabolism.

### Fruit diel changes are barely related to fruit photosynthesis or photorespiration but reveal links with the momentary supply of sucrose

Diel compositional changes in fruit were much lower than those observed during fruit development (Biais *et al.*, 2014), and were lower than diel changes in leaves, but several compounds expressed on a DW basis showed significant fluctuations. This difference compared with two previous studies on carbohydrates in apple (Klages *et al.*, 2001) and on hexoses and malate in tomato (Pearce *et al.*, 1992) may be due to differences in stages of development, culture conditions, or sampling. These diel changes result from *in situ* regulation of metabolism and/or source–sink relationships.

Concerning *in situ* metabolism, the photosynthesis of the green fruit contributes to its carbon balance (Smillie *et al.*, 1999) and its roles have been reconsidered recently (Cocaliadis *et al.*, 2014), but it does not seem to be involved in fruit diel changes. Indeed, in our study, the starch pattern in fruits for the control condition of Exp. 2 was in agreement with a transitory storage of carbon following sucrose import and also the limited contribution of *in situ* photosynthesis. However, no diurnal pattern was observed for sucrose in fruits, possibly due to an immediate use of both imported and *in situ*-produced sucrose for metabolism and growth. Among the compounds implicated in photorespiration, glycine and alanine did not show any diel pattern in fruits. This may be explained by the fact that the cell layers responsible for fruit photosynthesis (Smillie *et al.*, 1999) and photorespiration represent a limited portion of the tomato pericarp.

Starch accumulation characterizes the expansion phase of tomato fruit (Biais *et al.*, 2014). Starch and the adenosine-like compound peaked in the morning or at midday for the control condition of Exp. 2 and peaked latter in the afternoon for the shaded condition of this experiment. The conserved pattern of the adenosine-like compound peaking at midday in all experiments and conditions may be related to a tight regulation of the contents of nucleosides and nucleotides linked with co-enzyme pools. Targeted analyses of nucleosides, nucleotides, and phosphorylated intermediates in pericarp are needed to confirm this hypothesis. Tomato cell expansion is also characterized by anaplerosis, which enables the accumulation of organic and amino acids that provide the osmotic driving force (Biais *et al.*, 2014). In our study, several organic acids tended to accumulate at night in fruits: citrate, malate, and succinate in the control condition of Exp. 2, malate and succinate in the shaded condition of Exp. 2, and malate in the control condition of Exp. 1. This accumulation seems in agreement with a decreased fruit growth rate at night (Guichard *et al.*, 2005). Aspartate and glutamate also tended to accumulate at night in the pericarp in the control condition of Exp. 2, and this was maintained for glutamate for the shaded condition, possibly in relation to a decrease in protein synthesis at night (Pal *et al.*, 2013).

The fruit metabolite diel patterns may also provide clues for source–sink interactions. They were modified by carbon availability, as a range of amino acids showed diurnal patterns in the shaded condition of Exp. 2, and more phenolics were affected under the shaded condition of Exp. 1. Among the three major free amino acids of expanding tomato fruit, GABA, glutamine, and glutamate, the latter two compounds are the major amino acids transported by the phloem (Valle *et al.*, 1998). Whereas only aspartate and glutamate showed significant diel changes in fruits of the control and shaded conditions of Exp. 2, most other amino acids showed significant diel changes for the shaded condition of this experiment, all with a tendency to have higher levels at night. The major secondary metabolites observed in tomato green fruit are chlorogenic acid, α-tomatine, and dehydrotomatine (Friedman, 2002; Slimestad and Verheul, 2009). We observed a diel pattern for these two glycoalkaloids, peaking in the morning, for the control condition of Exp. 2. Labelling studies reported by Friedman (2002) showed that tomatine was synthesized in tomato fruit *de novo*, but translocation of alkaloids also seems possible (Lee *et al.*, 2007). Among phenolics, rutin was the only compound showing a diel pattern in fruit in at least two conditions. As for glycoalkaloids, this pattern may be linked to increased carbon availability for *in situ* synthesis in the fruit, as in poplar leaf (Arnold *et al.*, 2004), or to transitory influx changes after phloem transport. Indeed, the cellular transport of flavonoids has been reported, suggesting that import of phenylpropanoids from vegetative organs may exist (Zhao and Dixon, 2010). A metabolomics analysis of tomato phloem sap composition, including data about glycoalkaloids and phenolics, is therefore needed.

The diel patterns of several compounds were opposite in fruits compared with leaves. This was the case for malate and succinate in several conditions or experiments. Their higher content in leaves during the light period may be linked to the diel regulation of nitrogen assimilation (Tucker *et al.*, 2004) and the biosynthesis of amino acids exported in phloem. Their higher content in fruits at night may be linked to amino acid synthesis in fruit from the amino acids imported from phloem. Moreover, in Exp. 1 fruits, a range of amino acids tended to peak later in the shaded condition, possibly due to a lower carbon supply. This emphasizes the need to consider nitrogen partitioning in parallel with carbon partitioning to unravel source–sink relationships in tomato.

### Lower carbon availability increases metabolic co-ordination between leaf and fruit

Source–sink relationships were studied here by visualizing metabolic co-ordination between leaf and fruit. The partial correlation graphs showed that metabolic co-ordination between leaf and fruit during a diurnal cycle was higher for the shaded condition compared with the control condition. Another difference between the two conditions was the proportion of negative links on the partial correlation networks: about 10% in the control condition and 30% in the shaded one. This may reflect increased competition for carbon availability in the shaded condition, i.e. less competition for carbon availability in the control condition, in relation to a possible buffering for carbon availability through transitory storage in the stem for the latter condition, as proposed above. The hypotheses concerning metabolic co-ordination and competition for carbon could be tested using isotopic labelling experiments in order to study either the allocation and partitioning of major compounds or the regulation of metabolic fluxes.

Primary and secondary metabolites appeared within the compounds having a high number of connections in relation to the links between primary and secondary metabolism (Aharoni and Galili, 2011; Neilson *et al.*, 2013). Leaf trehalose, GABA, proline, succinate, and glycerol were linked to at least a fruit compound in both control and shaded conditions. The first three of these compounds are known to be key players. Trehalose may serve as a signalling molecule implicated in sugar sensing (Paul *et al.*, 2008), with a specific role for trehalose-6-phosphate. Trehalose has been reported in phloem sap in *Arabidopsis* (Hodge *et al.*, 2013). This allows us to hypothesize a possible link between leaf trehalose metabolism, possibly phloem transport, and fruit metabolism. GABA has also been proposed as a signal molecule, and the GABA shunt, involving succinate, has been proposed to play a major role in carbon and nitrogen primary metabolism (Fait *et al.*, 2008). Proline fulfils a variety of roles in plants, and its metabolism contributes to the redox balance (Szabados and Savoure, 2010). Fruit galactose, xylose, and kaempferol-3-rutinoside were linked to at least a leaf compound in both control and shaded conditions. Direct regulatory roles of these compounds are not documented. Galactose is one of the precursors of ascorbate. Xylose is a major component of tomato pericarp cell walls, since xyloglucan polysaccharides are the most abundant hemicellulose components in the primary cell walls of tomato. The metabolism of these hemicelluloses is key for wall loosening linked with cell expansion (Itai *et al.*, 2003). In the network of the shaded conditions, fruit xylose was connected to leaf trehalose. One might hypothesize a regulatory role for xylose too, possibly linking fruit expansion to leaf sugar sensing.

### Conclusions

Using a metabolomics approach, we confirmed the existence of diel patterns in tomato leaf composition. We also showed lower but significant diel changes in expanding tomato fruit with patterns that can be related to those of the closest leaf and depend on the potential carbon supply. Visualization of the co-regulations between compounds using correlation networks, and showing a higher proportion of negative correlations under shading than under optimal growth conditions, can be considered as a way to zoom in on the competition for carbon resources. Part of the results can be explained by sucrose supply with probable diel changes in phloem sap composition and/or flux, and also probable temporary carbohydrate storage in the stem that needs to be re-examined in detail. The present data will contribute towards pointing out unexpected phenotyping targets for the definition of ideotypes for tomato breeding or optimization of cultural practices.

## Supplementary data

Supplementary data are available at *JXB* online.

Supplementary text. Method details.

Supplementary Fig. S1. Fruit temperatures for the control and shaded conditions during the diel cycle of Exp. 2.

Supplementary Fig. S2. Variability of compound contents during a diel cycle. For each experiment and condition, distribution of the coefficients of variation of compounds measured in tomato leaf or fruit.

Supplementary Fig. S3. Heat maps of compound changes during the diel cycle for tomato leaf and fruit for the control and shaded conditions during the two experiments.

Supplementary Fig. S4. Diel changes in malate, succinate, aspartate, and glutamate contents measured in tomato fruit in Exp. 2 under the control or shaded condition.

Supplementary Fig. S5. Diel changes in sucrose, starch, amino acid, and protein contents measured in tomato fruit in Exp. 2 under the control or shaded condition.

Supplementary Table S1. Table of chemical shifts used for identification and quantification of metabolites in ^1^H-NMR spectra of extracts of tomato leaf and fruit.

Supplementary Table S2. List of metabolites putatively identified in tomato leaf and fruit extracts by LC-QTOF-MS analysis.

Supplementary Table S3. List of metabolites quantified in tomato leaf and fruit extracts by GC-TOF-MS analysis.

Supplementary Data

## Abbreviations:

ANOVA: analysis of variance
CV: coefficient of variation
DPA: days post-anthesis
DW: dry weight
FW: fresh weight
GABA: γ-aminobutyric acid
GC-MS: gas chromatography coupled with mass spectrometry
GGM: Gaussian graphical model
^1^H-NMR: proton NMR
LC-MS: liquid chromatography coupled with mass spectrometry
MS: mass spectrometry
PAR: photosynthetically active radiation
PCA: principal component analysis
SLA: specific leaf area
TOF: time of flight.
