# Supplementary material for: Metabolomic profiling in tomato reveals diel compositional changes in fruit affected by source–sink relationships
Source: J Exp Bot. 2015 Apr 11;66(11):3391–404. doi: 10.1093/jxb/erv151 (PMC4449552; doi:10.1093/jxb/erv151)
Supplement: Supplementary Data [file supp_66_11_3391__index.html]

Metabolomic profiling in tomato reveals diel compositional changes in fruit affected by source–sink relationships — Metabolomic profiling in tomato reveals diel compositional changes in fruit affected by source–sink relationships — Supplementary Data 

# Metabolomic profiling in tomato reveals diel compositional changes in fruit affected by source–sink relationships

## Supplementary Data

Data files

**Files in this Data Supplement:**

- Supplementary Data - Supplementary Data
- Supplementary Data - Supplementary Data
- Supplementary Data - Supplementary Data
