# Supplementary material for: Metabolomic profiling in tomato reveals diel compositional changes in fruit affected by source–sink relationships
Source: J Exp Bot. 2015 Apr 11;66(11):3391–404. doi: 10.1093/jxb/erv151 (PMC4449552; doi:10.1093/jxb/erv151)
Supplement: Supplementary Data [file supp_erv151_erv151_SI_TablesS1_2_3_Benard_JxB_finalpubpdf.pdf]

Journal of Experimental Botany
Metabolomic profiling in tomato reveals diel compositional changes in fruit affected by source-sink relationships
Camille Bénard, Stéphane Bernillon, Benoît Biais, Sonia Osorio, Mickaël Maucourt, Patricia Ballias, Catherine Deborde, Sophie Colombié, Cécile Cabasson, Daniel Jacob, Gilles Vercambre, Hélène Gautier, Dominique Rolin, Michel Génard, Alisdair R. Fernie, Yves Gibon, Annick Moing

Table S1: Table of chemical shifts used for identification and quantification of metabolites in ^{1}H-NMR spectra of polar extracts (in deuterated 250 or 125 mM phosphate buffer solution, apparent pH 6.0) of tomato fruit pericarp or mature leaf, expressed as relative values to the TSP resonance at 0 ppm. s: singlet, bs: broad singlet, d: doublet, dd: doublet of doublets, t: triplet, td: triplet of doublets, m: multiplet

Metabolites	Chemical shift (δ^1H in ppm D_2O pH 6) and Multiplicity	Chemical shift (δ^1H) of NMR signal used for quantification[c]	Identification status[a]	Tissue[b]
**Organic acids**				
Acetic acid	1.94 (s)	1.94	1	L
Citric acid	2.64 (dd)	2.64	1	L, P
Formic acid	8.46 (s)	8.46	2	L
Fumaric acid	6.52 (s)	6.52	2	L, P
Lactic acid	1.32 (d)	1.32	2	L
Malic acid	4.30 (dd), 2.68 (dd), 2.38 (dd)	4.30	1	L, P
Quinic acid	2.04 (d), 1.96 (m), 1.98 (m), 1.88 (dd)	1.96	1	L
Succinic acid	2.43 (s)	2.43	2	L, P
**Amino acids**				
Alanine	1.48 (d)	1.48	1	L, P
Asparagine	2.96 (dd), 2.87 (dd)	2.87	1	L, P
Aspartic acid	2.82 (dd)	2.82	1	L, P
GABA	3.02 (t), 2.30 (t), 1.91 (m)	2.30	1	L, P
Glutamic acid	2.36 (td), 2.10 (m)	2.36	1	L, P
Glutamine	2.456 (m), 2.14 (m)	2.456	1	L, P
Isoleucine	1.01 (d), 0.94 (t)	1.01	1	P
Leucine	0.96 (t)	0.96	2	P
Isoleucine + Leucine	0.94 (t), 0.96 (t)	0.94, 0.96	2	L
Phenylalanine	7.43 (m), 7.39 (m), 7.33 (m)	7.43, 7.39, 7.33	1	L, P
Proline	2.05 (m), 2.11 (m)	2.05	2	L, P
Threonine	d	1.33	2	L, P
Tyrosine	7.2 (d), 6.91 (d)	6.91	1	L, P
Valine	1.054 (d), 0.99 (d), 6.91 (d)	1.054	1	L, P

Table S1 continued

Metabolites	Chemical shift (δ^1H in ppm D_2O pH 6) and Multiplicity	Chemical shift (δ^1H) of NMR signal used for quantification[c]	Identification status[a]	Tissue[b]
**Sugars/ sugar alcohol**				
Fructose	4.12 (d), 4.04 (d), 4.00 (m), 3.91 (d), 3.88 (d), 3.81 (s), 3.79 (s), 3.725 s), 3.722 (d), 3.70 (s), 3.69 (d), 3.67 (bs), 3.66 (bs), 3.61 (bs), 3.58 (t)	4.12	1	L, P
α-Glucose	5.24 (d)d	5.24	1	L, P
β-Glucose	4.65 (d), 3.25 (dd)m	4.65	1	L, P
β-Galactose	4.58 (d)d	4.58	2	P
Inositol	3.28 (t), 3.535 (dd), 3.62 (t)s	3.28	1	L, P
Sucrose	5.42 (d), 4.22 (d), 4.04 (t), 3.68 (s), 3.47 (t), 3.765 (t), 3.82 (m)	5.42	1	L, P
**Other compounds**				
Choline	3.2 (s)	3.2	2	L, P
Trigonelline	9.13 (s), 8.845 (dd), 8.08 (t), 4.445 (s)	9.13	1	L, P
Adenosine-like	8.52 (s), 8.28 (s), 6.15 (d)	8.52	3	L, P
UDP-like	7.95 (d), 5.98 (m), 5.60 (m)	7.95	3	P
**Unknown compounds**				
UnknownD7.55	7.55 (d)	7.55	4	P
UnknownM6.35	6.35 (m)	6.35	4	L
UnknownM6.15	6.15 (m)	6.15	4	P
UnknownM5.83	5.837 (m)	5.837	4	P
UnknownM5.6	5.60 (m)	5.60	4	L
UnknownS5.40	5.40 (s)	5.40	4	P
UnknownD5.10	5.10 (d)	5.10	4	P
UnknownD5.06	5.06 (d)	5.06	4	L
UnknownD5.03	5.03(d)	5.03	4	L
UnknownS2.97	2.97 (s)	2.97	4	L, P
UnknownS2.76	2.76 (s)	2.76	4	P
UnknownS1.32	1.32 (s)	1.32	4	L, P
UnknownM1.08	1.08 (m)	1.08	4	P

Identification level according to MSI (Sumner *et al.*, 2007): 1, Identified compounds (checked with standard); 2, Putatively annotated compounds; 3, Putatively characterized compound classes; 4, Unknown.
[b] L, leaf; P, fruit pericarp.

Journal of Experimental Botany

Metabolomic profiling in tomato reveals diel compositional changes in fruit affected by source-sink relationships

Camille Bénard, Stéphane Bernillon, Benoît Biais, Sonia Osorio, Mickaël Maucourt, Patricia Ballias, Catherine Deborde, Sophie Colombié, Cécile Cabasson, Daniel Jacob, Gilles Vercambre, Hélène Gautier, Dominique Rolin, Michel Génard, Alisdair R. Fernie, Yves Gibon, Annick Moing

Table S2. List of metabolites putatively identified in tomato leaf and fruit methanolic extracts by LC-QTOF-MS analysis. Identification was done according to accurate m/z and comparison with data in Gomez-Romero *et al.* (2010) and Mintz-Oron *et al.* (2008).

**Metabolite Name** [a]	**Elemental composition**	**Retention Time** (min)	**Theoretical Mass** $[M+H]^{+b}$	**Measured mass** $[M+H]^+$	**m/z error** (ppm)	**Identification status** [c]	**Tissue** [d]
Adenosine	$C_{10}H_{13}N_5O_4$	1.1	268.1040	268.1040	-0.1	2	L
α-Tomatine	$C_{50}H_{83}NO_{21}$	12.9	1034.5530	1034.5468	-6.0	2	L,P
Chlorogenic-acid (5-*O*-caffeoylquinic acid)	$C_{16}H_{18}O_9$	10.4	355.1024	355.1016	3.7	2	L,P
Coumaric acid hexose or coumaroylhexose*	$C_{15}H_{17}O_8Na$*	10.3	349.0893	349.0890	-0.3	2	L,P
Cryptochlorogenic acid (3-*O*-caffeoylquinic acid)	$C_{16}H_{18}O_9$	11.0	355.1024	355.1000	-0.3	2	L,P
Dehydrotomatine	$C_{50}H_{81}NO_{21}$	12.7	1032.5374	1032.5296	-7.5	2	L,P
Kaempferol-3-*O*-rutinoside	$C_{27}H_{29}O_{15}$	13.2	595.1658	595.1604	-9.0	2	L,P
Unknown alkaloid	$C_{53}H_{80}NO_{20}$	11.6	1050.5268	1050.5241	-2.6	3	L
OH-Lycoperoside A or B or C	$C_{52}H_{85}NO_{24}$	12.1	1108.5534	1108.5428	-9.6	2	L,P
p-Coumaroyl derivative	$C_{14}H_{30}O_8$	10.0	327.2013	327.2009	-1.2	3	L
Rutin	$C_{27}H_{30}O_{16}$	12.4	611.1607	611.1593	-2.2	2	L,P
Rutin-pentoside	$C_{32}H_{37}O_{20}$	12.2	727.2080	727.2050	-4.1	2	L,P
Tomatine-like	$C_{60}H_{76}NO_{14}$	12.2	1034.5260	1034.5274	1.3	3	L
Tryptophan	$C_{11}H_{12}N_2O_2$	6.5	205.0972	205.0947	-12.0	2	L,P
Pantothenic acid	$C_9H_{17}NO_5$	5.5	220.1180	220.1175	-2.0	2	L,P
Pantothenic acid hexose	$C_{15}H_{27}NO_{10}$	7.8	382.1708	382.1713	1.4	2	L
Unk_679	-	14.2	-	679.2829	-	4	L, P
Unk_273	-	16.6	-	273.0774	-	4	L
Unk_284	-	3.1	-	284.0981	-	4	L

[a] Metabolite Name - Putative metabolite identification

[b] Theoretical Mass for $(M+H)^+$ - Theoretical monoisotopic mass calculated for the ion $(M+H)^+$.

[c] Identification level according to MSI (Sumner et al. 2007): 1, Identified compounds (checked with standard); 2, Putatively annotated compounds; 3, Putatively characterized compound classes; 4, Unknown.

[d] L, leaf blade; P, fruit pericarp.

*detected as sodium adduct only

References

Gomez-Romero M, Segura-Carretero A, Fernandez-Gutierrez A. 2010. Metabolite profiling and quantification of phenolic compounds in methanol extracts of tomato fruit. *Phytochemistry* **71:**1848-1864 DOI: 10.1016/j.phytochem.2010.08.002

Mintz-Oron S, Mandel T, Rogachev I, Feldberg L, Lotan O, Yativ M, Wang Z, Jetter R, Adato A, Aharoni A. 2008. Gene expression and metabolism in tomato fruit surface tissues. *Plant Physiology* **147**: 823-851. DOI: 10.1104/pp.108.116004

Journal of Experimental Botany

Metabolomic profiling in tomato reveals diel compositional changes in fruit affected by source-sink relationships

Camille Bénard, Stéphane Bernillon, Benoît Biais, Sonia Osorio, Mickaël Maucourt, Patricia Ballias, Catherine Deborde, Sophie Colombié, Cécile Cabasson, Daniel Jacob, Gilles Vercambre, Hélène Gautier, Dominique Rolin, Michel Génard, Alisdair R. Fernie, Yves Gibon, Annick Moing

Table S3. List of metabolites quantified in tomato leaf and fruit semi-polar extracts by GC-TOF-MS analysis.

**Metabolite Name** [a]	**Analyte** [b]	**Retention Time** (s)	**Tag Mass**	**Identification Status** [c]	**Tissue** [d]
Alanine, beta	Alanine, beta- (3TMS)	394.25	248	2	P,L
Dehydroascorbic acid	Dehydroascorbic acid dimer (TMS)	624.99	316	2	P
Erythritol	Erythritol (4TMS)	408.45	217	2	L
Fumaric acid	Fumaric acid (2TMS)	371.26	245	2	L,
Galactonic acid	Galactonic acid-1,4-lactone, D(-)- (4TMS)	618.87	217	2	L
Glyceric acid	Glyceric acid, DL- (3TMS)	345.05	189	2	L
Glycerol	Glycerol (3TMS)	291.78	205	2	L
Glycine	Glycine (3TMS)	325.18	174	2	L, P
Isoleucine	Isoleucine, L- (2TMS)	319.19	158	2	L
Lysine	Lysine, L- (4TMS)	615.47	156	2	L
Methionine	Methionine, DL- (2TMS)	474.33	176	2	L, P
Ornithine	Ornithine, DL- (4TMS)	570.43	142	2	L
Putrescine	Putrescine (4TMS)	517.18	174	2	L, P
Rhamnose	Rhamnose, DL- (1MEOX) (4TMS)	515.53	117	2	L, P
Serine	Serine, DL- (3TMS)	357.52	204	2	L, P
Threonine, allo	Threonine, allo-, DL- (3TMS)	368.81	218	2	L
Trehalose	Trehalose, alpha,alpha'-, D- (8TMS)	876.24	198	2	L
Tyramine	Tyramine (3TMS)	638.36	174	2	L
Xylose	Xylose, D- (1MEOX) (4TMS)	493.37	217	2	L, P

[a] Metabolite Name - Putative metabolite identification.
[b] Analyte chosen for relative quantification.
[c] Identification level according to MSI (Sumner et al. 2007): 1, Identified compounds (checked with standard); 2, Putatively annotated compounds; 3, Putatively characterized compound classes; 4, Unknown.
[d] L, leaf blade; P, fruit pericarp.
