# Supplementary material for: Metabolomic profiling in tomato reveals diel compositional changes in fruit affected by source–sink relationships
Source: J Exp Bot. 2015 Apr 11;66(11):3391–404. doi: 10.1093/jxb/erv151 (PMC4449552; doi:10.1093/jxb/erv151)
Supplement: Supplementary Data [file supp_erv151_jexbot143677_file001.pdf]

Journal of Experimental Botany

Metabolomic profiling in tomato reveals diel compositional changes in fruit affected by source-sink relationships

Camille Bénard, Stéphane Bernillon, Benoît Biais, Sonia Osorio[4], Mickaël Maucourt, Patricia Ballias, Catherine Deborde, Sophie Colombié, Cécile Cabasson, Daniel Jacob, Gilles Vercambre, Hélène Gautier, Dominique Rolin, Michel Génard, Alisdair R. Fernie, Yves Gibon, Annick Moing

Supplementary text: Material and Method details

Plant material and growth conditions

Tomato plants were grown in a greenhouse in South-West of France (44°23'56''N, 0°35'25''E) from June to September. The irradiance outside the greenhouse was recorded. In early June 2010, seedlings were transplanted onto rockwool blocks with a density of 2 plants m^{-2}. The nutrient solutions were adapted to plant growth, and the water supply was adjusted to the climate (at least 30% drainage, pH adjusted to 5.9, electrical conductivity to 2.2 mS cm^{-1}).

Ecophysiological modelling of carbon consumption rate

The fruit growth rate was calculated using the time-course curves of fruit dry weight. Data were fitted by a logistic function and the derivative of this function provided the fruit growth rate.

Fruit respiration rate and carbon consumption rate were calculated by using an ecophysiological process-based model (Jones *et al.*, 1991) were the carbon consumption rate is the sum of the assimilate needs for biomass synthesis, *i.e.* the growth rate and the respiration rate (Heuvelink, 1995).

$$\text{Carbon consumption rate} = \frac{dF_{DW}}{dt}\,(CDM + qg) + \mathrm{qm}\,F_{DW}.\,\mathrm{Q10}^{(\mathrm{T}-30)/10}$$

with F_{DW} the fruit dry weight (gDW $fruit^{-1}$), $\frac{dF_{DW}}{dt}$ the fruit growth rate (gDW $fruit^{-1}$ day^{-1}), CDM the carbon into biomass (0.415 gC gDW^{-1} measured in tomato pericarp), qg the growth respiration coefficient (qg =0.133 gC gDW^{-1}) and qm the maintenance coefficient (qm=0.004 gC gDW^{-1} day^{-1}), Q10=1.4 (Heuvelink, 1995), T the average fruit temperatures measured during Exp-2 cycle for shaded and control conditions respectively.

This ecophysiological model has been validated in several conditions (Dayan *et al.*, 1993; Dimokas *et al.*, 2009) and its sensitivity has been evaluated (Cooman and Schrevens, 2007). Similar results for the carbon consumption rate obtained by the ecophysiological model and the carbon imported by fruit obtained by a stoichiometric model have recently been shown ((Colombié *et al.*, 2015) Supporting_Information_Figure_S3).

Robotised analyses of metabolites and proteins

The sum of free amino acids, starch and protein contents were determined as previously described (Biais *et al.*, 2014). Aliquots of about 20 mg of fresh frozen powder were weighed in 1.1 mL Micronic™ tubes (Lelystad, The Netherlands) and stored at -80 °C until robotised analysis. Metabolites were extracted twice with 80% (v/v) ethanol and once with 50% ethanol (Geigenberger *et al.*, 1996). The sum of free amino acids was determined in the ethanolic supernatant. Starch and protein contents were determined in the pellet. Starch (Hendriks *et al.*, 2003), amino acids (Bantan-Polak *et al.*, 2001) and protein content (Bradford, 1976) were determined as previously described using 96-well polystyrene microplates (Sarstedt, Marnay, France). Extractions and assays were performed using a robotised Starlet platform (Hamilton, Villebon sur Yvette, France) and absorbencies were respectively read at 340 (carbohydrates) or 600 nm (protein content) in MP96 readers (SAFAS, Monaco). Fluorescamine fluorescence for amino acids was determined using a Xenius reader (SAFAS, Monaco) with excitation at 405 nm and detection at 485 nm.

^{1}H-NMR analyses

For ^{1}H-NMR profiling, polar metabolites, were extracted from the ground lyophilised samples as previously described (Biais *et al.*, 2009; Moing *et al.*, 2004) with minor modifications. Frozen powdered samples were lyophilised and polar metabolites were extracted from 20 mg of lyophilised powder with an ethanol-water series at 80°C. The supernatants were combined, dried under vacuum and lyophilised. Two technical replicates were prepared for each fruit sample, and one for each leaf sample. Each lyophilised extract was solubilised in 500 µL of 250 or 125 mM potassium phosphate buffer solution at apparent pH 6.0, for fruit or leaf samples respectively, 5 mM ethylene diamine tetraacetic acid disodium salt (EDTA), in D_2O, titrated with KOD solution to pH 6.00 ±0.02 when necessary, and lyophilised again. The lyophilised titrated extracts were stored in darkness under vacuum at room temperature, before ^{1}H-NMR analysis was completed within one week. Before ^{1}H-NMR analysis, 500 µL

of D_2O with sodium trimethylsilyl [2,2,3,3-d_4] propionate (TSP, 0.01% final concentration for chemical shift calibration) were added to the lyophilised titrated extracts. The mixture was centrifuged at 10,000 *g* for 5 min at room temperature. The supernatant was then transferred into a 5 mm NMR tube for acquisition. Quantitative ^{1}H-NMR spectra were recorded at 500.162 MHz and 300 K on a Bruker Avance III spectrometer (Wissembourg, France) using a 5-mm broadband inverse probe, a 90° pulse angle and an electronic reference for quantification (Biais *et al.*, 2009; Mounet *et al.*, 2007). The assignments of metabolites in the NMR spectra were made by comparing the proton chemical shifts with literature (Fan, 1996; Mounet *et al.*, 2007) or database values (MeRy-B 2011, http://bit.ly/meryb ; HMDB, http://www.hmdb.ca/), by comparison with spectra of authentic compounds recorded in the same solvent conditions (in-house library) and by spiking the samples. ^{1}H-^{1}H COSY NMR experiments were acquired for selected samples for assignment verification. For absolute quantification of metabolites, four calibration curves (glucose and fructose: 1.25 to 50 mM, glutamate and glutamine: 0 to 15 mM) were prepared and analysed under the same conditions. The glucose calibration was used for the absolute quantification of all compounds, as a function of the number of protons of selected resonances except fructose, glutamate and glutamine quantified using their respective calibration curve.

LC-QTOF-MS analyses

Twenty mg of lyophilized powdered sample were extracted with 1 mL of methanol/water 70/30 (v/v) with 0.1% formic acid and analysed as previously described (Pascual *et al.*, 2013) with minor modification. The extraction solvent was spiked with methyl vanillate as internal standard. The extraction was performed with an iced-cooled ultrasonic bath for 15 min. Extracts were then centrifuged and the supernatant was filtered before injection. Each sample was extracted twice as technological replicates. For each extract, 5 µL were injected twice into an Ultimate 3000 HPLC system (Dionex, Sunnyvale, USA) equipped with a C18 Gemini column (Phenomenex, Torrance, CA, USA) (2.0 x 150 mm, 3µm). Solvents A and B were H_2O with 0.1% (v) formic acid and acetonitrile, respectively. Flow rate was 350 µL/min. Separation gradient was 0-5 min 97%A; 5-30 min from 97%A to 5%A; 30-35 min 5%A; 35-36 min from 5%A to 97% A ; 36-51 min 97%A. MS detection was performed using a micrOTOF-Q (Bruker Daltonics, Bremen, Germany). The mass spectrometer was equipped with an ESI probe operated in positive mode. Spray voltage was set at 4.5 kV. Mass range was 50-1500 Da. The acquisition rate was 2 spectra.s^{-1}. MS spectra were *m/z*-calibrated using a 10 µM lithium formate solution. QC samples were constituted of one sample of the same

tissue and used after each set of 10 samples. Data were processed using QuantAnalysis (Bruker Daltonics, Bremen, Germany). Extracted ion chromatograms with an *m/z* window of +/- 0.3 were used to integrate peaks.

GC-TOF-MS analyses

Metabolites for GC-TOF-MS were extracted and analysed using a method described previously (Osorio *et al.*, 2012; Roessner *et al.*, 2001) with minor modifications. Thirty mg of fresh frozen powder were extracted in 1.4 mL methanol containing ribitol as internal standard. After incubation at 70°C for 15 min, the extract was centrifuged at 21,000 *g* for 10 min. To separate the polar and non-polar metabolites, the supernatant was carefully mixed with 750 µL chloroform and 1.5 mL water. A centrifugation step of 3,700 *g* separated the polar from the apolar phase. For further analysis, 150 µL of the upper (polar) phase were dried *in vacuo*. The pellet was resuspended in 40 µL of methoxyamine hydrochloride (20 mg mL^{-1} in pyridine) and derivatized for 2 h at 37°C. After the addition of 1 mL of *N*-methyl-*N*-[trimethylsilyl] trifluoroacetamide containing 20 µL of the retention time standard mixture of fatty acid methylesters, the mix was incubated at 37°C for 30 min. A volume of 1 µL of each sample was injected into a GC-TOF-MS system (Pegasus III, Leco, St Joseph, MI, USA) using an autosampler (PAL Agilent, Santa Clara, CA, USA). Helium acted as carrier gas at a constant flow rate of 2 mL s^{-1}. Gas chromatography was performed on a 30 m MDN-35 column (Supelco, Bellfonte, PA, USA). The injection temperature was 230°C; the transfer line and ion source were set at 250°C. The initial oven temperature (85°C) was constantly increased to a final temperature of 360°C by 15°C min^{-1}. After a delay of 180 s, mass spectra were recorded at 20 scans s^{-1} with 70–600 *m/z* scanning range.

Chemicals and Enzymes

For NMR analysis, D_2O (99.9%) was purchased from Eurisotop (Gif sur Yvette, France) and (trimethylsilyl) propionic-2,2,3,3-d4 acid (TSP) sodium salt (98%) from Aldrich (Saint Quentin Fallavier, France).

For LC-MS analysis, solvants and methyl vanillate were from Sigma-Aldrich (Saint Quentin Fallavier, France), acetonitrile was from Fischer Scientific (Illkirch, France). Ultrapure water was produced by a milliQ system (Millipore, Molsheim, France).

For GC-MS analyses, all chemicals and pure standard substances were purchased from either Sigma-Aldrich Chemie GmbH (Deisenhofen, Germany) or Merck KGaA (Darmstadt, Germany).

For robotised measurements, Bradford reagent, fluorescamine, HEPES, Tricine, Triton X100, and amyloglucosidase (E.C. 3.2.1.3) were purchased from Sigma-Aldrich (Lyon, France). NAD^+, $NADP^+$, α-amylase (E.C. 3.2.1.1), glucose-6P dehydrogenase grade II (E.C. 1.1.1.49), hexokinase (E.C. 2.7.1.1) and phosphoglucose isomerase (E.C. 5.3.1.9) were purchased from Roche (Meylan, France).

REFERENCES

Bantan-Polak T, Kassai M, Grant KB. 2001. A comparison of fluorescamine and naphthalene-2,3-dicarboxaldehyde fluorogenic reagents for microplate-based detection of amino acids. Analytical Biochemistry **297**, 128-136.

Biais B, Allwood JW, Deborde C, Xu Y, Maucourt M, Beauvoit B, Dunn WB, Jacob D, Goodacre R, Rolin D, Moing A. 2009. H-1 NMR, GC-EI-TOFMS, and Data Set Correlation for Fruit Metabolomics: Application to Spatial Metabolite Analysis in Melon. Analytical Chemistry **81**, 2884-2894.

Biais B, Bénard C, Beauvoit B, Colombié S, Prodhomme D, Ménard G, Bernillon S, Gehl B, Gautier H, Ballias P, Mazat J-P, Sweetlove L, Génard M, Gibon Y. 2014. Remarkable reproducibility of enzyme activity profiles in tomato fruits grown under contrasting environments provides a roadmap for studies of fruit metabolism. Plant Physiology **164**, 1204-1221.

Bradford MM. 1976. Rapid and sensitive method for quantitation of microgram quantities of protein utilizing principle of protein-dye binding. Analytical Biochemistry **72**, 248-254.

Colombié S, Nazaret, Bénard C, Biais B, Mengin V, Solé M, Fouillen L, Dieuaide-Noubhani M, Mazat J, Beauvoit B, Gibon Y. 2015. Modelling central metabolic fluxes by constraint-based optimization reveals metabolic reprogramming of developing tomato fruit. The Plant Journal **81**, 24-39.

Cooman A, Schrevens E. 2007. Sensitivity of the Tomgro model to solar radiation intensity, air temperature and carbon dioxide concentration. Biosystems Engineering **96**, 249-255.

Dayan E, van Keulen H, Jones JW, Zipori I, Shmuel D, Challa H. 1993. Development, calibration and validation of a greenhouse tomato growth model: I. Description of the model. Agricultural Systems **43**, 145-163.

Dimokas G, Tchamitchian M, Kittas C. 2009. Calibration and validation of a biological model to simulate the development and production of tomatoes in Mediterranean greenhouses during winter period. Biosystems Engineering **103**, 217-227.

Fan TWM. 1996. Metabolite profiling by one- and two-dimensional NMR analysis of complex mixtures. Progress in Nuclear Magnetic Resonance Spectroscopy **28**, 161-219.

Geigenberger P, Lerchl J, Stitt M, Sonnewald U. 1996. Phloem-specific expression of pyrophosphatase inhibits long-distance transport of carbohydrates and amino acids in tobacco plants. Plant Cell and Environment **19**, 43-55.

Hendriks JHM, Kolbe A, Gibon Y, Stitt M, Geigenberger P. 2003. ADP-glucose pyrophosphorylase is activated by posttranslational redox-modification in response to light and to sugars in leaves of Arabidopsis and other plant species. Plant Physiology **133**, 838-849.

Heuvelink E. 1995. Dry-matter production in a tomato crop - Measurements and simulation. Annals of Botany **75**, 369-379.

Jones JW, Dayan E, Allen LH, Van Keulen H, Challa H. 1991. A dynamic tomato growth and yield model (TOMGRO). Transactions of the ASABE **34**, 663–672.

Moing A, Maucourt M, Renaud C, Gaudillere M, Brouquisse R, Lebouteiller B, Gousset-Dupont A, Vidal J, Granot D, Denoyes-Rothan B, Lerceteau-Kohler E, Rolin D. 2004. Quantitative metabolic profiling by 1-dimensional H-1-NMR analyses: application to plant genetics and functional genomics. Functional Plant Biology **31**, 889-902.

Mounet F, Lemaire-Chamley M, Maucourt M, Cabasson C, Giraudel JL, Deborde C, Lessire R, Gallusci P, Bertrand A, Gaudillere M, Rothan C, Rolin D, Moing A. 2007. Quantitative metabolic profiles of tomato flesh and seeds during fruit development: complementary analysis with ANN and PCA. Metabolomics **3**, 273-288.

Osorio S, Do PT, Fernie AR. 2012. Profiling primary metabolites of tomato fruit with gas chromatography/mass spectrometry. Methods in Molecular Biology **860**, 101-109.

Pascual L, Xu J, Biais B, Maucourt M, Ballias P, Bernillon S, Deborde C, Jacob D, Desgroux A, Faurobert M, Bouchet JP, Gibon Y, Moing A, Causse M. 2013. Deciphering genetic diversity and inheritance of tomato fruit weight and composition through a systems biology approach. Journal of Experimental Botany **64**, 5737-5752.

Roessner U, Willmitzer L, Fernie AR. 2001. High-resolution metabolic phenotyping of genetically and environmentally diverse potato tuber systems. Identification of phenocopies. Plant Physiology **127**, 749-764.
