# Supplementary material for: Metabolomic profiling in tomato reveals diel compositional changes in fruit affected by source–sink relationships
Source: J Exp Bot. 2015 Apr 11;66(11):3391–404. doi: 10.1093/jxb/erv151 (PMC4449552; doi:10.1093/jxb/erv151)

Journal of Experimental Botany

Metabolomic profiling in tomato reveals diel compositional changes in fruit affected by source-sink relationships

Camille Bénard, Stéphane Bernillon, Benoît Biais, Sonia Osorio, Mickaël Maucourt, Patricia Ballias, Catherine Deborde, Sophie Colombie, Cécile Cabasson, Daniel Jacob, Gilles Vercambre, Hélène Gautier, Dominique Rolin, Michel Génard, Alisdair Fernie,Yves Gibon, Annick Moing

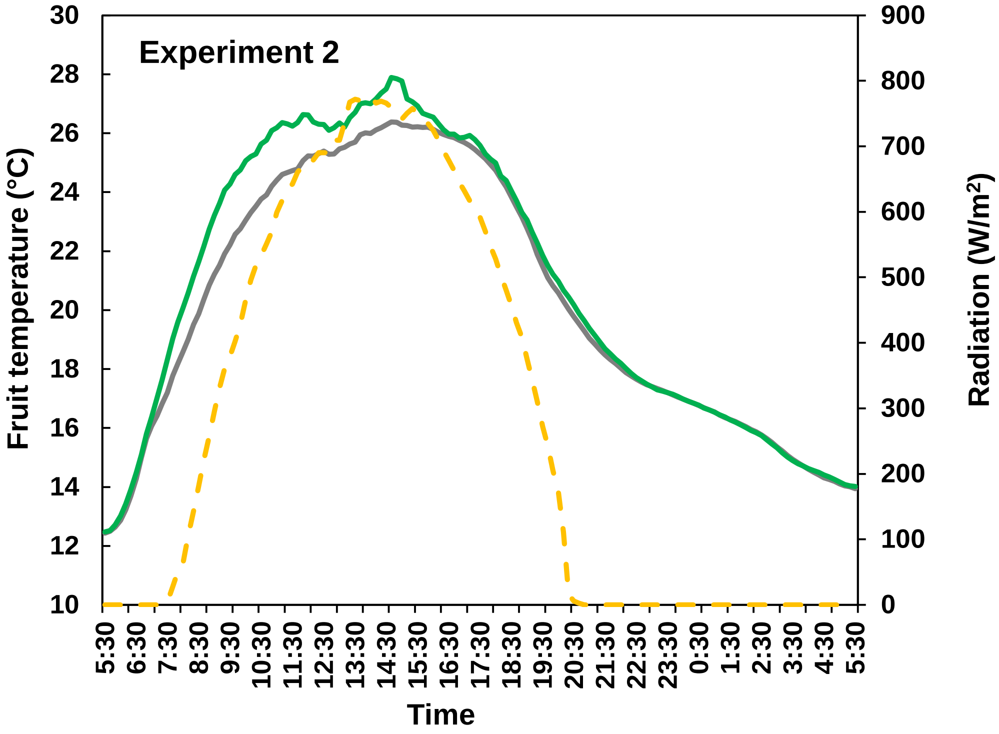

Figure S1. Fruit temperatures for the control and shaded conditions during the diel cycle of Experiment 2 (sunny day in August). Mean of 4 fruits per condition with one thermocouple per fruit. Control condition, green. Shaded condition, grey. The irradiance outside the greenhouse (yellow dotted line) is added to show the photoperiod.

Journal of Experimental Botany

Metabolomic profiling in tomato reveals diel compositional changes in fruit affected by source-sink relationships

Camille Bénard, Stéphane Bernillon, Benoît Biais, Sonia Osorio, Mickaël Maucourt, Patricia Ballias, Catherine Deborde, Sophie Colombie, Cécile Cabasson, Daniel Jacob, Gilles Vercambre, Hélène Gautier, Dominique Rolin, Michel Génard, Alisdair Fernie,Yves Gibon, Annick Moing

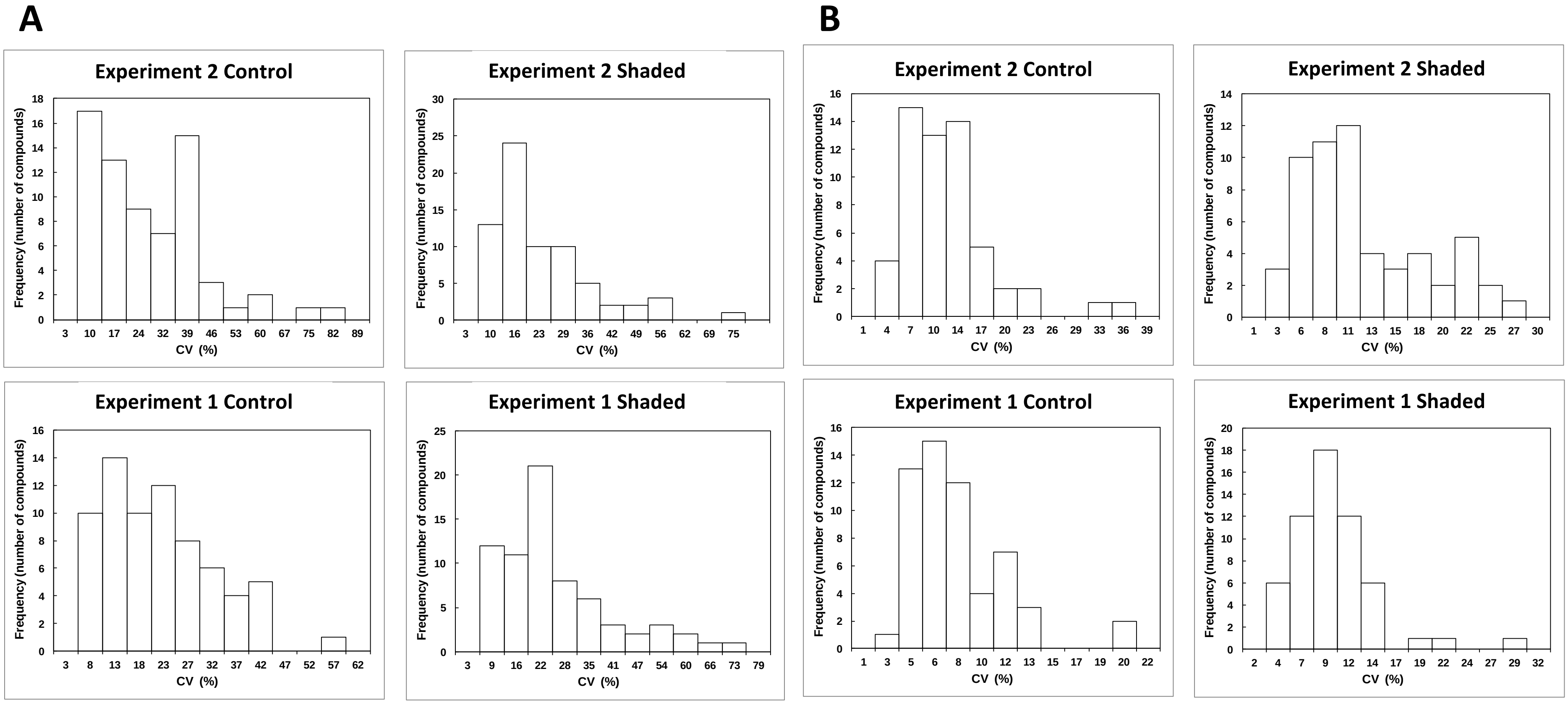

Figure S2. Variability of absolute or relative contents of metabolites during a diel cycle. For each experiment and condition, distribution of the coefficients of variation of 70 metabolites measured in mature leaf (A), or 57 metabolites in expanding fruit (B), harvested at 7 times with 4 replicates at each time, except for the shaded condition of Experiment 2 with 5 times and 4 replicates.

Figure S3: Heat maps of compound changes during the diel cycle for tomato mature leaf and expanding fruit for the control and shaded condition during the two experiments. Means of the biological replicates for each time. Time points superior to 24:00 correspond to the following day. Data normalization: mean centering and reduction to unit variance. A. Leaf in control condition for Experiment 1. B. Leaf in shaded condition for Experiment 1. C. Leaf in control condition for Experiment 2. D. Leaf in shaded condition for Experiment 2 . E. Fruit in control condition for Experiment 1. F. Fruit in shaded condition for Experiment 1. G. Fruit in control condition for Experiment 2. H. Fruit in shaded condition for Experiment 2.
* indicates a significant time effect according to one-factor ANOVA for each condition per experiment (*P*<0.05).

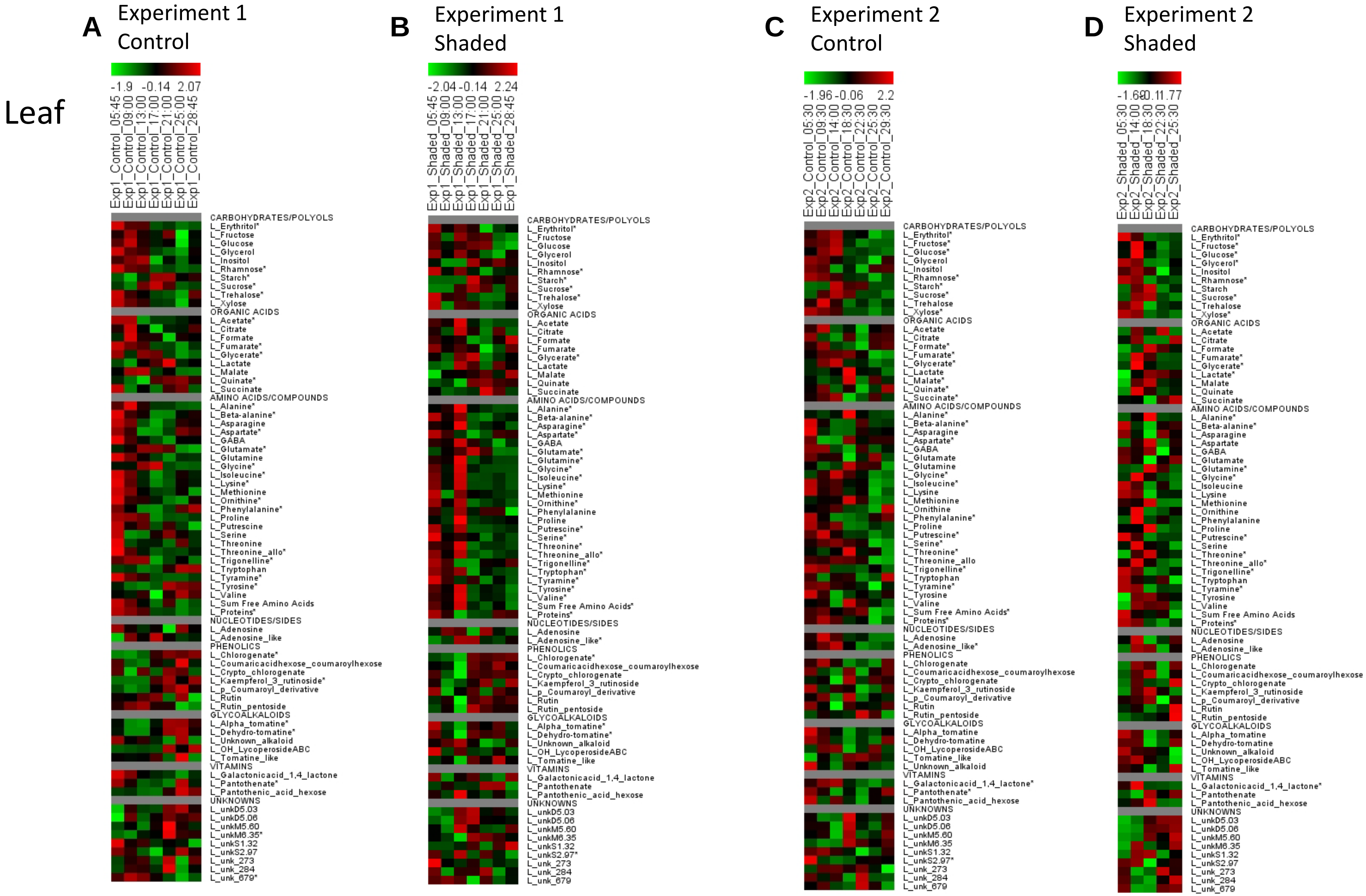

Figure S3: Heat maps of compound changes during the diel cycle for tomato mature leaf and expanding fruit for the control and shaded condition during the two experiments. Means of the biological replicates for each time. Time points superior to 24:00 correspond to the following day. Data normalization: mean centering and reduction to unit variance. A. Leaf in control condition for Experiment 1. B. Leaf in shaded condition for Experiment 1. C. Leaf in control condition for Experiment 2. D. Leaf in shaded condition for Experiment 2. E. Fruit in control condition for Experiment 1. F. Fruit in shaded condition for Experiment 1. G. Fruit in control condition for Experiment 2. H. Fruit in shaded condition for Experiment 2.

* indicates a significant time effect according to one factor ANOVA for each condition per experiment (*P*<0.05).

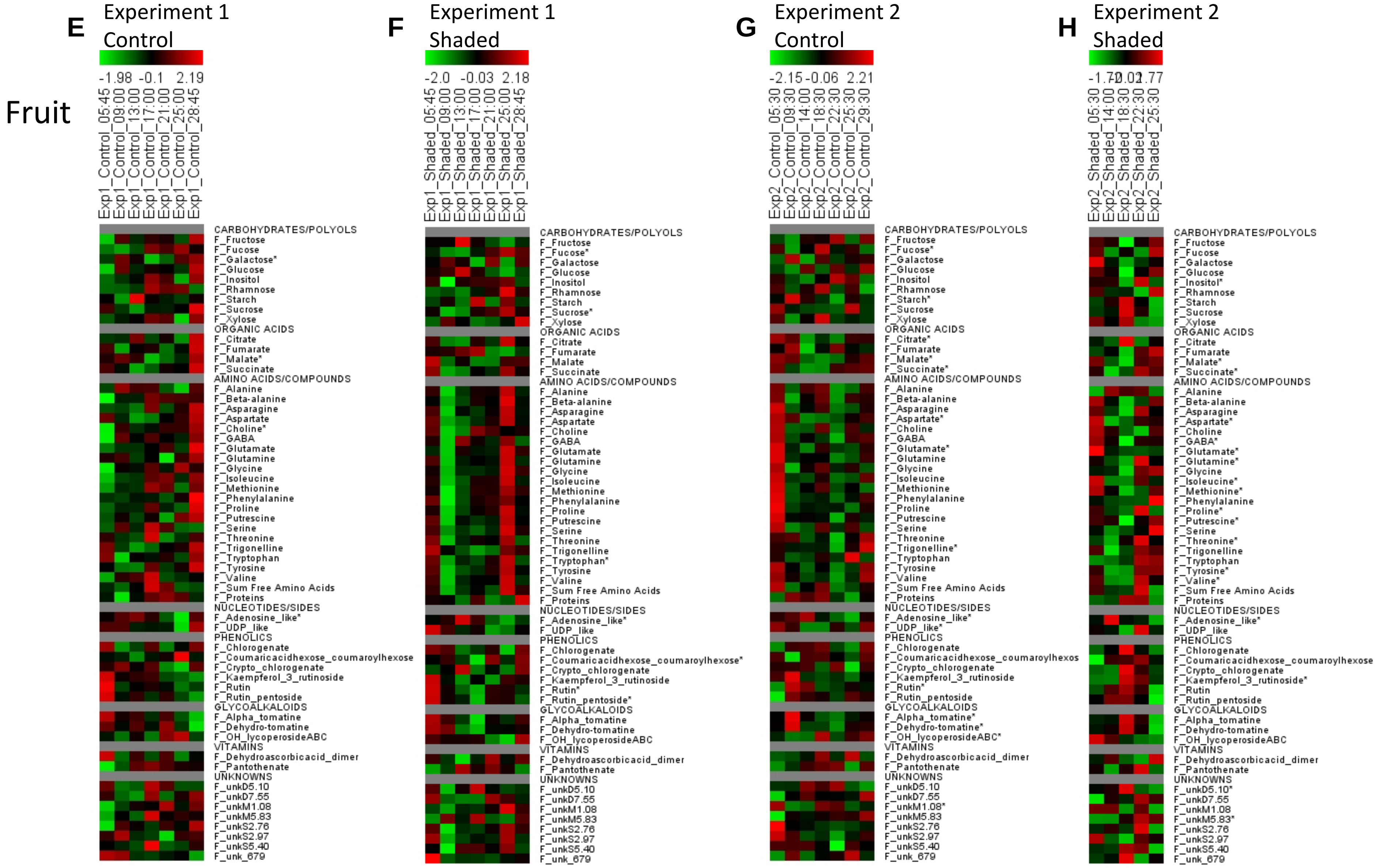

Journal of Experimental Botany

Metabolomic profiling in tomato reveals diel compositional changes in fruit affected by source-sink relationships

Camille Bénard, Stéphane Bernillon, Benoît Biais, Sonia Osorio, Mickaël Maucourt, Patricia Ballias, Catherine Deborde, Sophie Colombie, Cécile Cabasson, Daniel Jacob, Gilles Vercambre, Hélène Gautier, Dominique Rolin, Michel Génard, Alisdair Fernie,Yves Gibon, Annick Moing

Figure S4: Diel changes in malate, succinate, aspartate and glutamate contents measured in pericarp of expanding tomato fruit in Experiment 2, under the control (black) or shaded (grey) condition. Mean of 4 replicates. Vertical bars represent standard deviation.

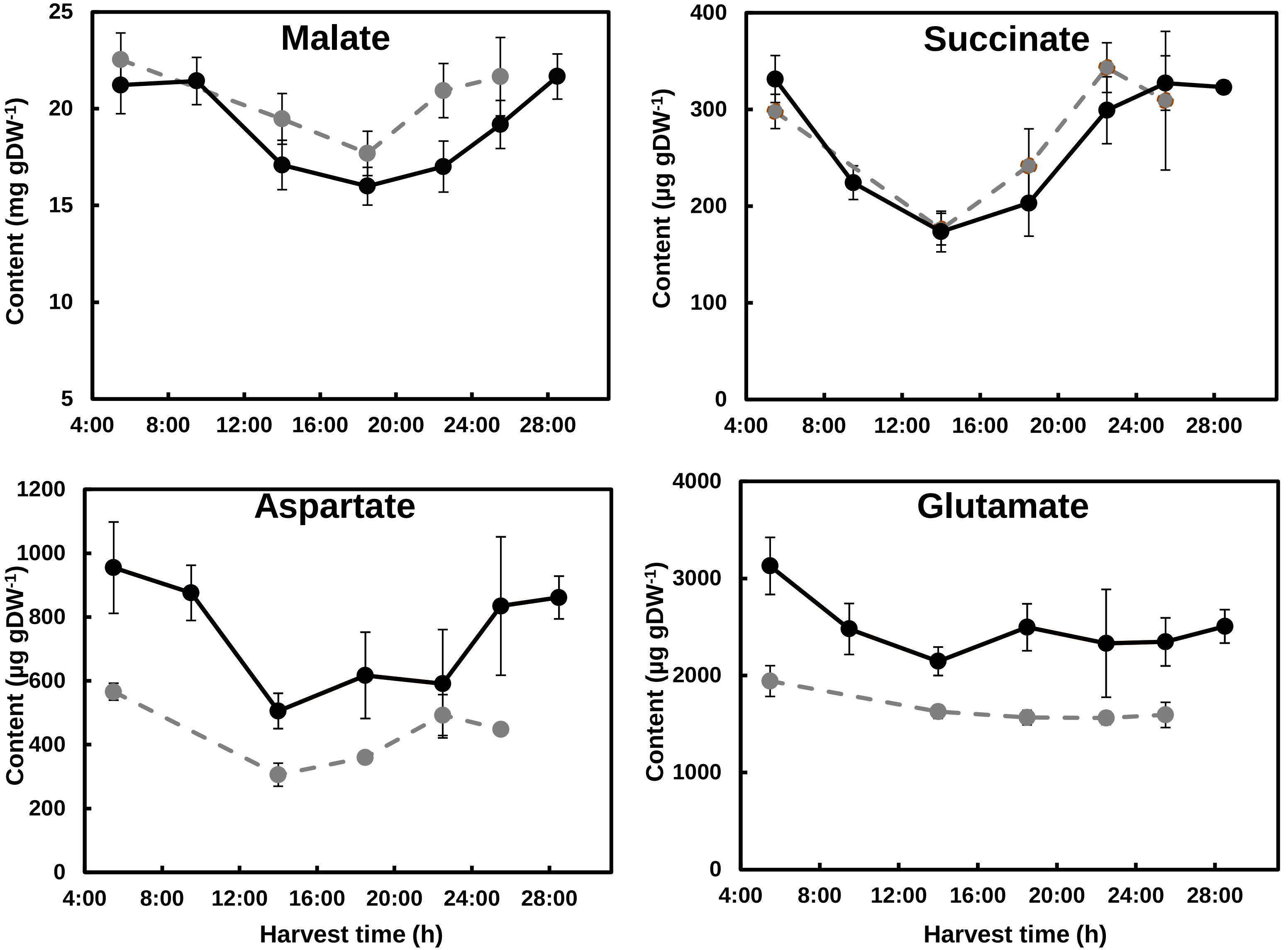

Journal of Experimental Botany

Metabolomic profiling in tomato reveals diel compositional changes in fruit affected by source-sink relationships

Camille Bénard, Stéphane Bernillon, Benoît Biais, Sonia Osorio, Mickaël Maucourt, Patricia Ballias, Catherine Deborde, Sophie Colombie, Cécile Cabasson, Daniel Jacob, Gilles Vercambre, Hélène Gautier, Dominique Rolin, Michel Génard, Alisdair Fernie,Yves Gibon, Annick Moing

Figure S5: Diel changes in sucrose, starch, sum of amino acids (sum of alanine, asparagine, aspartate, GABA, glutamine, glutamate, isoleucine, phenylalanine, proline, threonine, tyrosine, and valine determined with ^{1}H-NMR) and protein contents measured in pericarp of expanding tomato fruit in Experiment 2, under the control (black) or shaded (grey) condition. Mean of 4 replicates. Vertical bars represent standard deviation.

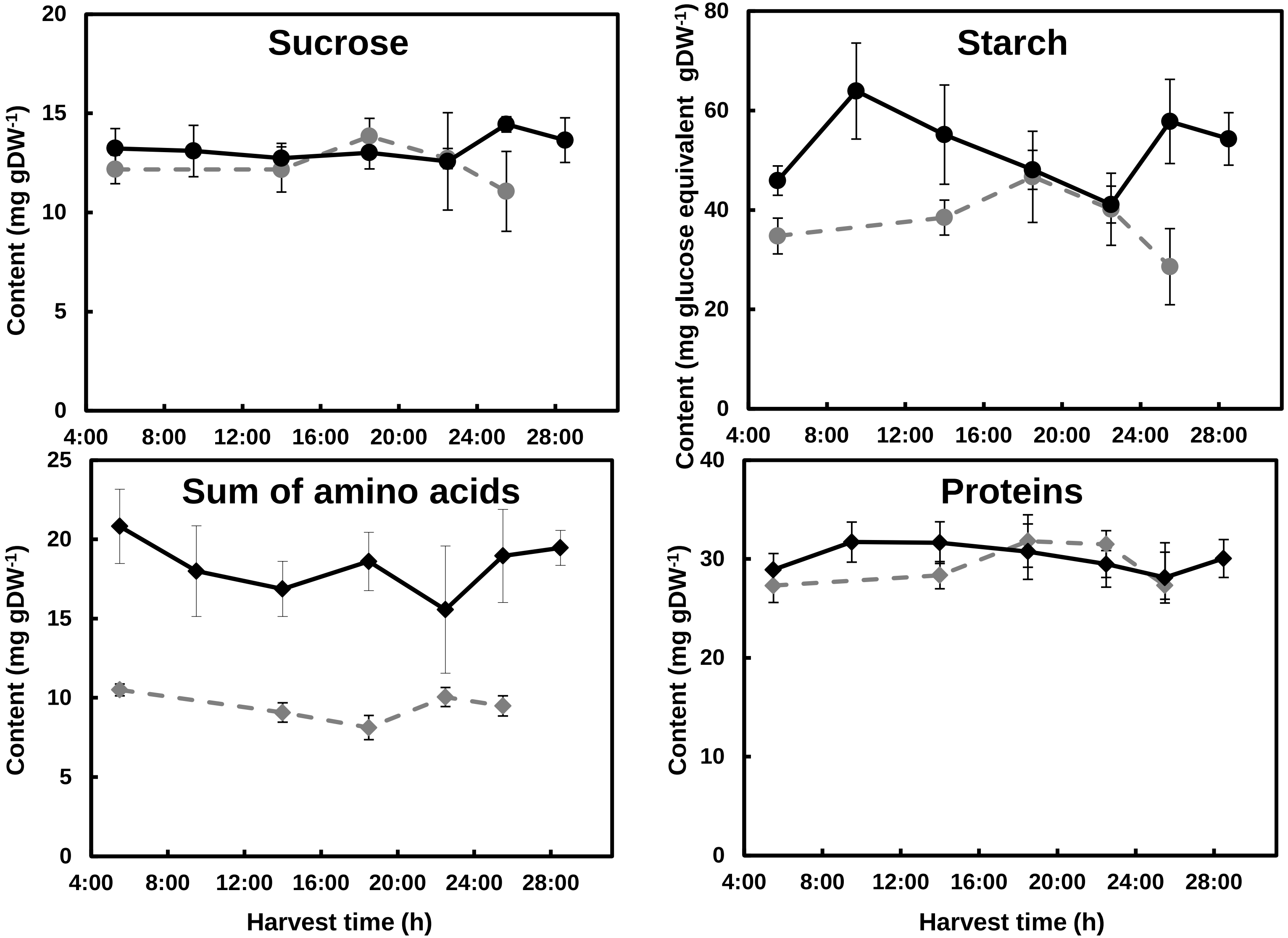

Supplement: Supplementary Data [file supp_erv151_jexbot143677_file002.pdf]
